# Correction: Analysis of Response Elements Involved in the Regulation of the Human Neonatal Fc Receptor Gene (*FCGRT*)

**DOI:** 10.1371/journal.pone.0139744

**Published:** 2015-09-29

**Authors:** Joanna E. Mikulska

## Notice of Republication

This article was republished on September 17, 2015, to correct formatting errors to Tables 1–3 and symbol errors in figure legends. Please download this article again to view the correct version. The originally published, uncorrected article and the republished, corrected article are provided here for reference.

## Supporting Information

S1 File Originally published, uncorrected article. (PDF)

S2 File Republished, corrected article. (PDF)
