# Supplementary material for: Correction: Analysis of Response Elements Involved in the Regulation of the Human Neonatal Fc Receptor Gene (FCGRT)
Source: PLoS One. 2015 Sep 29;10(9):e0139744. doi: 10.1371/journal.pone.0139744 (PMC4587857; doi:10.1371/journal.pone.0139744)
Supplement: S1 File — (PDF) [file pone.0139744.s001.pdf]

PLOS | ONE

RESEARCH ARTICLE

Analysis of Response Elements Involved in the Regulation of the Human Neonatal Fc Receptor Gene (*FCGRT*)

Joanna E. Mikulska*

Department of Immunochemistry, Ludwik Hirszfeld Institute of Immunology and Experimental Therapy, Polish Academy of Sciences, Wroclaw, Poland

* mikulska@iitd.pan.wroc.pl

CrossMark
click for updates

OPEN ACCESS

Citation: Mikulska JE (2015) Analysis of Response Elements Involved in the Regulation of the Human Neonatal Fc Receptor Gene (*FCGRT*). PLoS ONE 10 (8): e0135141. doi:10.1371/journal.pone.0135141

Editor: Sebastian D. Fugmann, Chang Gung University, TAIWAN

Received: February 10, 2015

Accepted: July 19, 2015

Published: August 7, 2015

Copyright: © 2015 Joanna E. Mikulska. This is an open access article distributed under the terms of the Creative Commons Attribution License, which permits unrestricted use, distribution, and reproduction in any medium, provided the original author and source are credited.

Data Availability Statement: All relevant data are within the paper and its Supporting Information files.

Funding: This work was supported by Polish Ministry of Science and Higher Education/National Science Centre (grant N N407 173434). The authors thank the Wrocław Centre of Biotechnology, The Leading National Research Centre (KNOW) programme for years 2014–2018 for covering the publication fee. The funders had no role in study design, data collection and analysis, decision to publish, or preparation of the manuscript.

Competing Interests: The author has declared that no competing interests exist.

Abstract

Human epithelial, endothelial and PMA-differentiated THP-1 cell lines were used as model systems to study the transcriptional regulation of the human *FCGRT* gene encoding the alpha chain of hFcRn. The data obtained from site-directed mutagenesis in transient transfection experiments indicate that the Sp1 sites at positions -641, -635, and -313, CF1/YY1 elements at positions -586 and -357, and the AP-1 motif at -276 within the-660/-233 fragment of the human *FCGRT* promoter (h*FCGRT*) participate in the regulation of human *FCGRT* in all selected cell lines. However, their individual contribution to promoter activity is not equivalent. The Sp1 binding site at -313 and the AP-1 site at -276 are critical for the activity of the h*FCGRT* promoter in epithelial and endothelial cells. Moreover, the CF1/YY1 site at -586 in differentiated THP-1 cells, plays an essential role in the transcriptional activity of the promoter. In addition, the C/EBPbeta binding site at -497 of the h*FCGRT* promoter in epithelial and endothelial cells, and the C/EBPbeta motif located at -497 and -233 within the h*FCGRT* promoter in differentiated THP-1 cells may function as positive regulatory sequences in response to LPS or PMA stimulation. EMSA and supershift analyses showed that the functionally identified binding motifs in the h*FCGRT* promoter were able to specifically interact with their corresponding (Sp1, Sp2, Sp3, c-Fos, c-Jun, YY1, and C/EBPbeta or C/EBPdelta) transcription factors (TFs), suggesting their possible involvement in the regulation of the human *FCGRT* gene expression.

Introduction

The neonatal Fc receptor, FcRn, was originally identified as a receptor responsible for the IgG binding to the intestinal epithelium and fetal yolk sack of neonatal rats and mice [1–3]. The human homologue of the rodent FcRn (hFcRn) was first cloned from a human placental cDNA library [4]. Subsequently, hFcRn was identified in many fetal and adult human tissues of all ages [4–9] and various cell types, including epithelial cells [10,11], endothelial cells [12,13], macrophages, dendritic cells [14] and neutrophils [15]. The hFcRn receptor is

structurally related to the MHC class I proteins and consists of transmembrane α-chain (45 kDa) in noncovalent association with β2-microglobulin (light chain, 12 kDa) [16]. This receptor binds the Fc domain of IgG at pH 6.0–6.5, but at pH 7.0–7.4 it shows low or no affinity [12]. This pH-dependent hFcRn-Fc(IgG) interaction is responsible for transcytotic and protective functions of hFcRn. The human neonatal Fc receptor is a key protein involved in the transport of maternal IgG to the fetus, providing protective immunity to the newborn [17]. The second important role of hFcRn is the protection of IgG from catabolism and the maintenance of IgG and albumin homeostasis [18–20]. This Fc receptor can also extent the life span of autoimmune IgG [21]. Recent studies have also shown that hFcRn may play an important role in mucosal immunity [22,23] and can modulate antigen presentation by dendritic cells [24,25]. On account of the vital roles of the hFcRn receptor in the protection and transportation of IgG under normal and inflammatory situations, there is a great need to elucidate the mechanism that controls the expression of the human *FCGRT* gene. A more complete understanding of this gene regulation will provide a possibility to modulate the biological functions of hFcRn, with potential future applications, for example in the therapy of IgG-mediated autoimmune diseases. Knowledge of the exon/intron organization and sequence of the gene encoding human FcRn α chain (*FCGRT)* [26], as well as the promoter activity of the 5′-flanking sequence of human *FCGRT* [27] provided a starting point for investigating the regulation of expression of this gene at the transcriptional level.

Previous analysis of the promoter region of the human *FCGRT* gene suggested the involvement of Sp1 and AP-1 elements in the control of promoter activity; however, a detailed analysis of these sites in terms of controlling the expression of this gene has not been performed [27]. This study applied electrophoretic mobility shift assay, antibody supershift analysis, and site-directed mutagenesis in transient transfection experiments to examine whether the potential regulatory elements within a fragment (-660/-233) of the h*FCGRT* promoter are involved in the transcriptional regulation of the human *FCGRT* gene in human epithelial and endothelial cells and differentiated THP-1 cells. The data revealed that the Sp1, AP-1, CF1/YY1 *cis* elements in the -660 to -233 sequence participate in the transcriptional regulation of human *FCGRT* in epithelial, endothelial and PMA-differentiated THP-1 cell lines. Furthermore, the C/EBPβ binding site at -497 within the h*FCGRT* promoter in Caco-2, Lu 106 and HSkMEC cells, and the C/EBPβ motif located at -497 and -233 in the h*FCGRT* promoter in THP-1 cells, may function as positive regulators of this gene transcription under stimulated conditions.

Materials and Methods

Materials

Dulbecco's modified Eagle's medium, RPMI 1640 medium, Minimum Essential Medium alpha (MEM-α) were obtained from the Laboratory of Biopreparations of the Institute of Immunology and Experimental Therapy (Wroclaw, Poland). Tissue culture plates were purchased from Costar (USA). Bacterial LPS from *E. coli* (serotype 055:B5 and serotype 0127:B8), L-glutamine, antibiotics (penicillin/streptomycin mixture), non-essential amino acids, phorbol 12-myristate 13 acetate, F-12 Ham medium, heparin, normal human serum, oligo(dT)$_{23}$ primers, poly(dI-dc)poly(dI-dc), protease inhibitor cocktail, and phosphatase inhibitor cocktail were obtained from Sigma-Aldrich (USA). Fetal bovine serum (FBS) and Opti-MEM I reduced serum medium were purchased from Gibco BRL (UK). RNaseOUT recombinant ribonuclease inhibitor and Superscript III Reverse Transcriptase were purchased from Invitrogen (USA). HyClone fetal bovine serum was acquired from Thermo Scientific, Inc. QIAquick Gel Extraction Kit and QIAquick Nucleotide Removal Kit were obtained from Qiagen GmbH (Germany). TransIT-2020 transfection reagent was purchased from Mirus Bio (USA). Restriction enzymes were

obtained from New England BioLabs (USA). Rabbit polyclonal antibodies against Sp1 (PEP2), Sp2 (K-20), Sp3 (D-20), c-Fos, (K-25), c-Jun (D), YY1 (C-20), C/EBPβ (C-19), and C/EBPδ (M 17) were obtained from Santa Cruz Biotechnology (USA). Plasmid Midi AX Kit and Plasmid Mini Kit for DNA plasmid purification and Gel-Out DNA fragment purification kit were purchased from A&A Biotechnology (Poland). Dual-luciferase reporter assay system reagents were acquired from Promega GmbH (Germany). DreamTaq DNA polymerase, dNTP mix, T4 polynucleotide kinase, T4 DNA ligase, GeneRuler 1kb DNA Ladder, and GeneRuler 100 bp DNA Ladder Plus were obtained from MBI Fermentas (Lithuania). FCGS was purchased from BD Biosciences. Biocoat Collagen I Cellware plates and flask were purchased from Becton Dickinson (UK). QuickChange Lightning Site-Directed Mutagenesis Kit was obtained from Stratagene (USA). Labeled [gamma ^{32}P]ATP was obtained from Hartmann Analytic GmbH (Germany). NucleoSpin RNA II kit was acquired from Macherey-Nagel GmbH (Germany).

Cell culture

The human colon epithelial cell line Caco-2, human monocyte cell line THP-1, and human promyelocytic leukemia cell line HL-60 were obtained from the American Type Culture Collection (ATCC). The Caco-2 cells were grown in Dulbecco's modified Eagle's medium supplemented with 12% fetal bovine serum (FBS), 0.03% L-glutamine, 100 units/ml penicillin, 100 g/ml streptomycin, and 1% non-essential amino acids. THP-1 cells were cultured in RPMI 1640 medium supplemented with 10% FBS, 0.03% L-glutamine, 25 mM HEPES, 1 mM sodium pyruvate, 100 units/ml penicillin, and 100 g/ml streptomycin. To induce monocytes to macrophage differentiation, THP-1 cells were grown in the presence of 10 ng/ml PMA for 3 days. The Lu 106 epithelium-like cell line, human umbilical vein endothelial cell line (HUVEC), and human skin microvascular endothelial cell line (HSkMEC) [28] were purchased from the Cell Lines Collection at the Institute of Immunology and Experimental Therapy (Wroclaw, Poland). The human cell lines Lu 106 and HL-60 were propagated in Minimum Essential Medium (MEM-α) supplemented with 10% FBS, 0.03% L-glutamine, 100 units/ml penicillin, and 100 g/ml streptomycin. HUVEC were cultured in medium F12 supplemented with 10% HyClone FBS, 100 units/ml penicillin, 100 g/ml streptomycin, 0.03% L-glutamine, 0.1 mg/ml heparin, and 0.03 mg/ml FCGS. HSkMEC cells were grown in Opti-MEM I Reduced Serum Medium supplemented with 5% HyClone FBS, 100 units/ml penicillin, and 100 g/ml streptomycin. All cells were maintained at 37°C and 5% CO_2 in a humidified incubator. In the PMA stimulation experiments, HSkMEC, Lu 106 and Caco-2 cells were cultured in growth medium with 5% FBS in the presence of 100 ng/ml PMA for 6 hours. For the LPS treatment, these cells were incubated in medium supplemented with 5% normal human serum containing 5 g/ml LPS (*Escherichia coli* 055:B5) for 1 or 6 hours.

Reverse transcription (RT)-PCR

Total RNA was isolated from cells (2 x 10^6) using the NucleoSpin RNA II kit according to the manufacturer's recommendations. First-strand cDNA was synthesized from total RNA using SuperScript III Reverse Transcriptase and an oligo(dT)$_{23}$ primer as recommended by the manufacturer. PCR was performed using the following mixture: 25 u/ml Taq DNA polymerase (DreamTaq) in the buffer provided, 1.5 mM $MgCl_2$, 0.2 mM dNTP mix, 0.2 μM concentrations of each primer and 2 μl cDNA in a 20 μl final volume. PCR was initiated with a denaturing step at 94°C for 3 min followed by 35 amplification cycles (25 for *GAPDH*) consisting of a denaturing step at 94°C for 1 min (30 s for *GAPDH*), annealing at 58°C for 1 min (55°C, 30 s for *GAPDH*), and extension at 72°C for 1 min. At the end of the 35 cycles (25 for *GAPDH*),

reaction was run for an additional 10 min at 72°C and then maintained at 4°C until analyzed by 1.5% agarose gel electrophoresis.

The following primers specific for human *FCGRT* were used: FcRn1 (sense: 5′-CTCTCCCT CCTGTACCACCTT-3′, α1 domain); FcRn2 (antisense: 5′-ATAGCAGGAAGGTGAGCTCC T-3′, α2 domain). The cDNA was also amplified by *GAPDH*-specific primers (sense: 5′-ATGA CATCAAGAAGGTGGTG-3′; antisense: 5′-CATACCAGGAAATGAGCTTG-3′) as a control to monitor the quality of the RNA purification and cDNA synthesis. The PCR products for human *FCGRT* were extracted from the gel and purified using the QIAquick Gel Extraction Kit and then sequenced by GENOMED S.A. (Warsaw, Poland).

Preparation of nuclear extracts

Nuclear extracts were prepared from: PMA-differentiated THP-1 cells, differentiated THP-1 after stimulation with LPS, Lu 106, Caco-2 and endothelial cell lines (untreated and treated either with PMA or LPS), according to the method of Schreiber et al. [29] with minor modifications. Briefly, the collected cells were washed twice with cold PBS, and then suspended in ice-cold lysis buffer A (10 mM HEPES pH 7.9, 10 mM KCl, 0.1 mM EDTA, 0.1 mM EGTA, 1 mM DTT, 0.25% Nonidet P-40, 10 μl/ml protease inhibitor cocktail, 10 μl/ml phosphatase inhibitor cocktail 2), and placed on ice for 15 min. After centrifugation at 14,000 rpm for 5 min at 4°C, supernatants were removed and the nuclear pellets were washed once with ice-cold buffer A and then resuspended in three volumes of ice-cold buffer B (20 mM HEPES pH 7.9, 10 mM KCl, 420 mM NaCl, 0.1 mM EDTA,0.1 mM EGTA, 1 mM DTT, 20% glycerol, 10 μl/ml protease inhibitor cocktail), and gently vortexed in a cold room for 30 min. Cell debris was removed by centrifugation at 14,000 rpm for 20 min at 4°C and the supernatants (nuclear extracts) were collected and stored at -80°C in small aliquots until used in electrophoretic mobility shift assays. Protein concentration in nuclear extracts was determined by the Bradford protein assay [30].

Electrophoretic mobility shift assay (EMSA)

To prepare the double-stranded DNA oligonucleotides (wild-type and its mutated version) for EMSA, single-stranded forward and reverse oligonucleotides were annealed by heating to 95°C and cooling slowly to room temperature in annealing buffer (10 mM Tris-HCl, pH 7.5, 1 mM $MgCl_2$, 50 mM NaCl). Wild-type ds-oligonucleotides (double-stranded oligonucleotides) were employed either as probes following the 5′-end labeling using [γ-^{32}P]ATP and T4 polynucleotide kinase, or as unlabeled competitors. The mutated ds-oligonucleotides were only used as competitors. Single-stranded oligonucleotides (wild type and mutated) were synthesized and purified on HPLC by GENOMED S.A. (Warsaw, Poland). They represented the sequences of the h*FCGRT* promoter encompassing the putative transcription factor binding motifs. The sense sequences of the oligonucleotides used in EMSA are shown in Table 1.

DNA binding reactions were performed by preincubating the nuclear extract (10–15 μg of protein) with 2 μg of poly(dI-dC)·poly(dI-dC) in binding buffer (10 mM Tris-HCl, pH 7.5, 50 mM NaCl, 1 mM $MgCl_2$, 0.5 mM EDTA, 0.5 mM DTT, 4% glycerol) for 15 min on ice followed by the addition of approximately 70 fmol of ^{32}P-labeled probe and an additional 30 min incubation at room temperature. In competition experiments, a 100-fold molar excess of unlabeled competitor (wild type or mutant ds-oligonucleotide) was preincubated with the extract before the addition of the labeled probe. For supershift assays, nuclear extract in binding buffer (without DTT) was preincubated with a rabbit polyclonal antibody against the transcription factor or non-immune rabbit IgG for 60 min on ice, and then with poly(dI-dC)·poly(dI-dC) for 15 min prior to incubation with the radiolabeled probe. Two microgram of antibody was used in

Table 1. Sense sequences of the oligonucleotides used in EMSA. Mutated nucleotides are indicated in lowercase letters. WT – wild-type oligonucleotide. m – mutant oligonucleotide.

Name	Sequence 5′ → 3′
AP1 (WT) mAP1	TACAGGCGTGAGTCACTGCGCCCGGCCCGCA TACAGGCGaGctcCACTGCGCCCGGCCCGCA
CF1-1(WT) mCF1-1	ATGGGGACCATGTGGTCACTGAAGTCCTA ATGGGGAttcTGcGGTCACTGAAGTCCTA
CF1-2(WT) mCF1-2	TGTCACCATATTGGCCAGGCTGGTCT TGTCAttcTATTGGCCAGGCTGGTCT
C/EBPβ-1(WT) mC/EBPβ-1	AGTGGTGCAATCTCGGCTCACTGCGA AGTGGTaaccaCTCGGCTCACTGCGA
C/EBPβ-2(WT) mC/EBPβ-2	GAGCACCTGCAGGAATTTTTTAAGGGGATG GAGCACCTatcaGTcTTTTTTAAGGGGATG
Sp1-1+2(WT) mSp1-2 mSp1-1+2	GAGTGTGGGCGGGGGCTGGTGCCTGGAGGGGCCTTC GAGTGTGGGCGGGGGtctGTGCCTGGAGGGGCCTTC GAGTGTGttgGGGGGtctGTGCCTGGAGGGGCCTTC
Sp1-3(WT) mSp1-3	TGATCCGCCCGCCTTGGCCTCCCAAAGT TGATCCGCCCtaCTTGGCCTCCCAAAGT

doi:10.1371/journal.pone.0135141.t001

each supershift assay. Subsequently, the samples were resolved on 5% polyacrylamide gels in 0.5 x TBE buffer (45 mM Tris-borate, 1 mM EDTA, pH 8.3). Electrophoresis was performed at 180 V for 2 h at room temperature. After electrophoresis, the gels were autoradiographed by exposure to storage phosphor screens for 24–72 h at -20°C, and the DNA-protein complexes were analyzed using a phosphor imager (Typhoon 8600) equipped with ImageQuant software (Molecular Dynamics).

Construction of promoter-luciferase plasmids

The DNA fragment (bases -660 to +52) of the h*FCGRT* promoter was released from the pGEM-2400 plasmid (containing the 5′-flanking region of the human *FCGRT* gene) with *Kpn*I and *Avr*II. Then this fragment was subcloned into the *Kpn*I/*Nhe*I sites of the promoterless firefly luciferase reporter vector, pGL3-basic, to obtain the wild type h*FCGRT* promoter reporter construct, designated as pGL3-711(WT). The orientation and nucleotide sequence of the DNA fragment (-660/+52) inserted into the pGL3-basic luciferase vector was verified by sequencing (GENOMED S.A., Warsaw, Poland). Mutant promoter constructs were generated from the pGL3-711(WT) plasmid using the QuickChange Lightning Site-directed Mutagenesis Kit, according to the manufacturer's recommendations. Primer sets used to create the desired mutations in the transcription factor binding sites within the fragment (-660/+52) of the h*FCGRT* promoter are listed in Table 2. The reaction mixture was composed of 5 μl of 10 x reaction buffer, 50 ng of DNA template, 125 ng of primers, 1 μl of dNTP mix, 1.5 μl of Quick-Solution reagent, and 1 μl of QuickChange Lightning enzyme in a final volume of 50 μl. The reaction was initiated with a denaturing step at 95°C for 2 min, followed by PCR amplification with denaturation at 95°C for 20 s, annealing at 60°C for 10 s, extension at 68°C for 2 min and 36 s for a total of 18 cycles, and final extension at 68°C for 5 min. Subsequently, the reaction mixture was cooled on ice and 1 μl of DpnI was added to digest the original DNA template for 5 min at 37°C. The PCR products were then transformed into XL-10-Gold ultracompetent cells. The resulting promoter-luciferase plasmids were designated as follows: pGL3-711(mSp1-1), pGL3-711(mSp1-2), pGL3-711(mSp1-3), pGL3-711(mSp1-1+2), pGL3-711(mAP1), pGL3-711(mCF1-1), pGL3-711(mCF1-2), pGL3-711(mC/EBPβ-1) and pGL3-711(mC/EBPβ-2). Each individual mutant promoter construct carried a mutation in a single consensus binding site. Three binding motifs for Sp family transcription factors, two for CF1/YY1 family nuclear proteins, and two for the C/EBP family were identified within the -660/-233 fragment of the *hFCGRT* promoter. Therefore, mutant promoter constructs were also generated, carrying mutations in all binding sites specific for a given transcription factor. The pGL3-711(mSp1-1+2) mutant construct served as a template to obtain the pGL3-711(mSp1-1+2+3) mutant construct containing the mutated Sp1 binding sites at positions -641, -635 and -313. The

Table 2. Oligonucleotide pairs used for making the mutant luciferase reporter constructs.

Name	Sequencechange	Sequence[a] Sense primer (5′ to 3′) Antisense primer (5′ to 3′)	Position[b]
mSp1-1	GGC to ttg	**GAGTGTGttgGGGGGCTGGTGCCTGGAGGGGCCTTC**GAAGGCCCCTCCAGGCACCAGCCCCCcaaCACACTC	-646/-611
mSp1-2	CTG to tct	**GAGTGTGGGCGGGGGtctGTGCCTGGAGGGGCCTTC**GAAGGCCCCTCCAGGCACagaCCCCCGCCCACACTC	-646/-611
mSp1-1+2	GGC to ttg CTG to tct	_________GAGTGTGttgGGGGGtctGTGCCTGGAGGGGCCTTC**GAAGGCCCCTCCAGGCACagaCCCCCcaaCACACTC**	-646/-611
mSp1-3	GC to ta	**TGATCCGCCCtaCTTGGCCTCCCAAAGT**ACTTTGGGAGGCCAAGtaGGGCGGATCA	-320/-293
mAP1	T to a AGT to ctc	**TACAGGCGaGctcCACTGCGCCCGGCCCGCA**TGCGGGCCGGGCGCAGTGgagCtCGCCTGTA	-284/-254
mCF1-1	CCA to ttc, T to c	**ATGGGGAttcTGcGGTCACTGAAGTCCTA**TAGGACTTCAGTGACCgCAgaaTCCCCAT	-593/-565
mCF1-2	CCA to ttc	**TGTCAttcTATTGGCCAGGCTGGTCT**AGACCAGCCTGGCCAATAgaaTGACA	-362/-337
mC/EBPβ-1	GCAAT to aacca	**AGTGGTaaccaCTCGGCTCACTGCGA**TCGCAGTGAGCCGAGtggttACCACT	-499/-474
mC/EBPβ-2	GCAG to atca, AA to tc	**GAGCACCTatcaGtcTTTTTAAGGGGATG**CATCCCCTTAAAAAgaCtgatAGGTGCTC	-240/-211

[a] Putative transcription factor binding sites are underlined and mutated nucleotides are indicated in lowercase letters. Single-stranded oligonucleotides were synthesized by Genomed S.A.

[b] Position of oligonucleotides relative to the transcriptional start site (+1).

doi:10.1371/journal.pone.0135141.t002

pGL3-711(mC/EBPβ-1) construct, carrying a mutation in the C/EBPβ site at position -497 was used to generate the pGL3-711(mC/EBPβ-1+2) reporter construct containing mutations in both C/EBPβ motifs (at -497 and -233). The pGl3-711(mCF1-1) construct with a mutation of the CF1/YY1 site at position -586 served as a template to create the pGL3-711(mCF1-1+2) construct carrying mutations in CF1/YY1 binding sites located at positions -586 and -357. The promoter reporter constructs were purified using Plasmid Midi AX Kit and all mutations were confirmed by DNA sequencing.

Transient transfections and luciferase assays

Transient transfections of all cell types were carried out in 12-well tissue culture plates using T*rans*IT-2020 reagent as described by the manufacturer. Briefly, the day before transfection, Caco-2, Lu106 and HSkMEC cells were seeded at a density of 1.9–2.3 x 10^5 cells/well, and THP-1 at 1 x 10^6 cells/well, in 1 ml of complete growth medium. Directly before transfection, the cells were washed twice with PBS and once with the complete growth medium without antibiotics and incubated in 1 ml of the same medium for 1–2 h. DNA complexes with T*ransIT*-2020 reagent were prepared as follows: 0.9–1.8 μg/well of promoter luciferase construct, or the promoterless luciferase pGL3-basic plasmid (negative control), plus 0.1–0.2 μg/well of *Renilla* luciferase vector (phRG-B) in 100 μl of Opti-MEM I reduced serum medium were gently mixed with T*rans*IT-2020 reagent. The ratio of T*rans*IT-2020 reagent to DNA was optimized for each cell type. After incubation of the mixtures at room temperature for 20 min, generated complexes were added to the wells with cells (100 μl of each complex into one well) and incubated at 37°C in 5% CO_2. Twenty-four hours after transfection, the cells were washed twice with PBS and lysed in 250 μl/well of 1x passive lysis buffer. The lysates were centrifuged at 3000 g for 5 min at room temperature to remove cell debris and then the supernatants were assayed for luciferase activity. The transfected THP-1 cells were exposed to PMA (100 ng/ml) for 4 hours prior to lysis. Firefly and *Renilla* luciferase activities in 20 μl of cell lysate was measured according to the Dual Luciferase Reporter Assay System protocol using a Glomax 20/20 luminometer. In each experiment, the firefly luciferase activity from various promoter luciferase constructs and pGL3-basic plasmid was normalized to the *Renilla* luciferase activity driven by the cotransfected phRG-B vector. The normalized transcriptional activity of the mutant constructs and pGl3-basic plasmid was presented as a percentage of the normalized activity of the wild-type pGL3-711(WT) construct, which was defined as 100%.

Statistical analysis

Data from transient transfection assays are presented as means ± SD of three to six independent experiments performed in duplicate for each construct. Statistical analysis of the results was carried out using two-way analysis of variance (ANOVA) as well as Student's *t* test.

Results

Expression of human *FCGRT* mRNA in human cell lines

For studies on the regulation of transcription of human *FCGRT*, human epithelial and endothelial cell lines and PMA-differentiated THP-1 cells were used. These cell lines were chosen, because they represent cell types that have been found to express hFcRn *in vivo*. Although hFcRn was previously reported to be expressed in THP-1 cells [14] and the human intestinal Caco-2 cell line [31], the presence of the human *FCGRT* mRNA was verified in the selected Caco-2, Lu 106, HUVEC, HSkMEC, and THP-1 cell lines by RT-PCR to make sure that these cells are a suitable model systems for studying transcriptional regulation of human *FCGRT*.

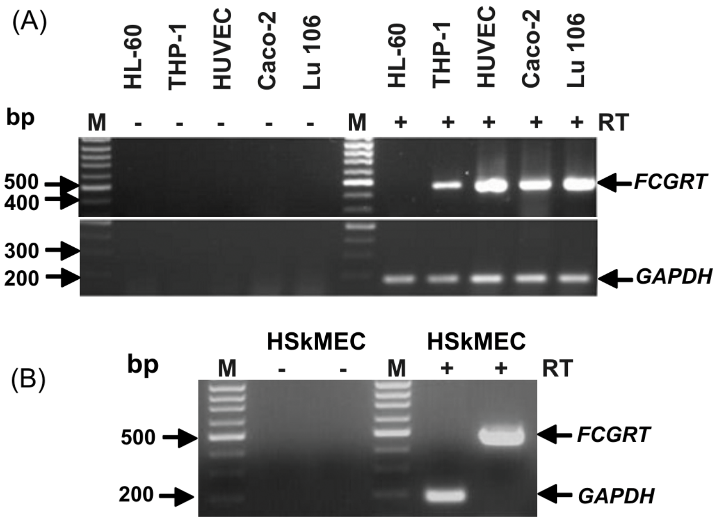

Fig 1. RT-PCR analysis of human *FCGRT* mRNA in cell lines: (A) THP-1, HUVEC, Caco-2, and Lu 106, (B) HSkMEC. Total RNA from these cell lines was subjected toRT-PCR using primers specific for human *FCGRT* and *GAPDH*. For control samples, reverse transcription was omitted. The amplified PCR products with (+) or without (-) reverse transcription (RT) were analyzed by 1.5% agarose gel electrophoresis and stained with ethidium bromide. Arrows indicate the location of the amplification products of expected sizes for human *FCGRT* and *GAPDH*. The size marker-GeneRuler-100 bp DNA ladder (M). Total RNA from the HL-60 cell line was subjected to RT-PCR analysis (as negative control, *FCGRT* mRNA is not expressed in this cell line).

doi:10.1371/journal.pone.0135141.g001

The results of RT-PCR screening are shown in Fig 1, where the PCR product of the expected size of 457 bp is clearly visible. Sequencing of this PCR product demonstrated 100% identity with the previously reported sequence of the human *FCGRT* gene [26].

EMSA and antibody supershift analysis of the putative transcription factor binding sites in the h*FCGRT* promoter

Two types of computer programs (MatInspector and Transfac) were used to identify putative binding motifs for transcription factors within the h*FCGRT* promoter, and sites selected to the study on the transcriptional regulation of the human *FCGRT* gene are shown in Table 3. The potential transcription factor binding elements listed in Table 3 are located in the -660/-233 region of the promoter, which is essential for its activity [27], most of the selected elements was also found in the 5′-flanking region of the mouse *Fcgrt* gene [32].

To test whether the potential regulatory elements interact with nuclear extracts (from the selected cell lines), and to identify the transcription factors specifically binding to these

Table 3. Potential transcriptional regulatory elements in the -660/-233 fragment of the h*FCGRT* promoter.

Name	Consensus sequence	Position[a]
AP-1	TGAGTCA	-276
CF1/YY1	CCATGT CCATAT	-586 -357
C/EBPβ	TGGTGCAAT TGCAGGAAT	-497 -233
Sp1	TGGGCGGGG GGGGCTGGT CCCGCC	-641 -635 -313

[a] Numbers refer to the first base of the putative transcription factor binding site. Positions of the potential transcriptional regulatory motifs are relative to the transcription start site (+1) of the human *FCGRT* gene.

doi:10.1371/journal.pone.0135141.t003

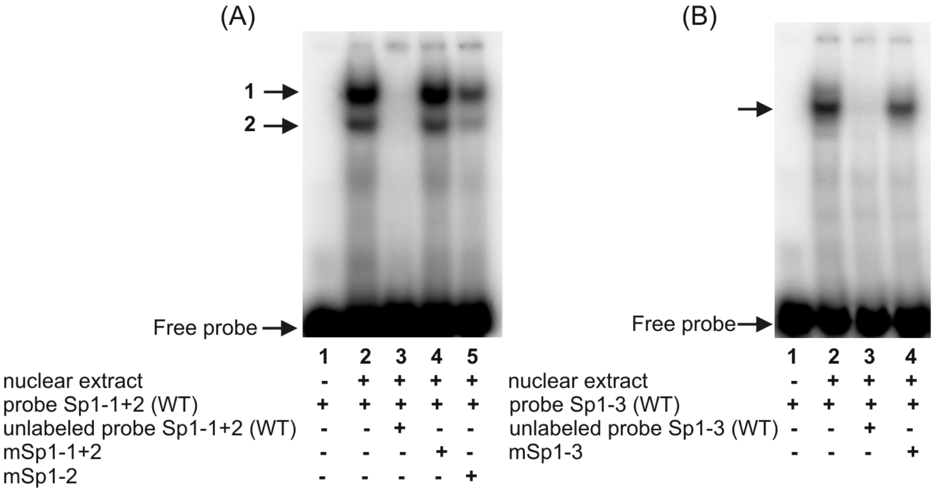

Fig 2. Electrophoretic mobility shift assays with probe Sp1-1+2 (A) and probe Sp1-3 (B). Probe Sp1-1 +2 – double-stranded oligonucleotide representing the -646/-611 sequence of the h*FCGRT* promoter, containing the potential transcriptional regulatory Sp1 motifs at positions -641 and -635; probe Sp1-3 – ds-oligonucleotide corresponding to the sequence -320/-293 of the h*FCGRT* promoter containing a putative Sp1 binding site at position -313. Probes were end-labeled with [γ^{32}P]ATP] and incubated with nuclear extract (12 μg) in the absence of competitor (A and B, lanes 2) or in the presence of a 100-fold molar excess competitor: Sp1-1+2(WT)–unlabeled wild-type probe Sp1-1+2 (A, lane 3), Sp1-3(WT)–unlabeled wild-type probe Sp1-3 (B, lane 3), mSp1-1+2 – unlabeled probeSp1-1+2 containing mutation in the Sp1 sequence at positions -641 and -635 (A, lane 4), mSp1-2 – unlabeled probe Sp1-1+2 containing mutation in the Sp1 sequence at position -635 (A, lane 5), mSp1-3 – unlabeled probe Sp1-3 containing mutation in the Sp1 sequence at position -313 (B, lane 4). Labeled probe Sp1-1+2(WT) alone (A, lane 1), labeled probe Sp1-3 (WT) alone (B, lane 1). DNA-protein complexes were resolved on 5% non-denaturing polyacrylamide gels and analyzed in a phosphor imager (Typhoon 8600) using ImageQuant software (Molecular Dynamics). Positions of specific DNA-protein complexes are indicated by arrows.

doi:10.1371/journal.pone.0135141.g002

regulatory motifs, EMSA and antibody supershift assays were performed. Double-stranded oligonucleotides for these experiments were prepared by annealing the complementary single-stranded oligonucleotides representing sequences of the h*FCGRT* promoter encompassing the putative transcription factor binding motif. Wild-type ds-oligonucleotides used as probes in EMSA and supershift assays were 5′-end labeled using [γ^{32}P]ATP and T4 polynucleotide kinase.

Representative results from EMSA and supershift tests of the h*FCGRT* promoter are presented in Figs 2–5, Figs 6A and 7. They were obtained using the nuclear extract from PMA-differentiated THP-1 cells. A similar or identical banding pattern was observed with nuclear extracts from epithelial and endothelial cells (Fig 6B–6D, S1–S4 Figs).

Incubation of nuclear extracts with radiolabeled ds-oligonucleotide Sp1-1+2, containing putative partly overlapping Sp1 binding motifs located at -641 and -635, produced two DNA-protein complexes (Fig 2A, lane 2). A single band was revealed in reaction with ds-oligonucleotide Sp1-3 containing a putative binding site at position -313 (Fig 2B, lane 2). These complexes were abolished when nuclear extracts were preincubated with a 100-fold molar excess of unlabeled wild-type oligonucleotides (Fig 2A and 2B, lanes 3), but not the mutant oligonucleotides (Fig 2A, lanes 4,5 and 2B, lane 4). The competition results indicated that nuclear proteins present in the selected cell lines interacted specifically with GC box at position -641, -635 and -313. To determine the nature of the transcription factor interacting with the identified regulatory Sp1 elements within the h*FCGRT* promoter, supershift assays were performed with antibodies against the Sp family transcription factors. The upper complex 1 supershifted with antibodies specific for Sp1 (Fig 3A, lane 4) and Sp2 (Fig 3A, lane 5). Antibody against Sp3 caused a loss of

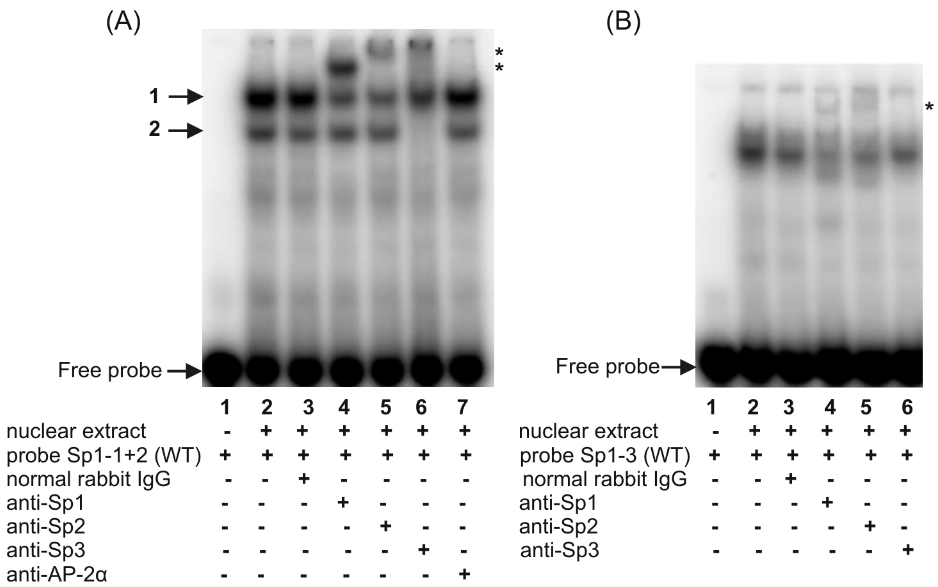

Fig 3. Identification of transcription factors binding to the Sp1 sequences within the -660/-233 fragment of the h*FCGRT* promoter. Supershift experiments were performed by preincubating the nuclear extract from differentiated THP-1 cells on ice for 1 h with 2 g of rabbit polyclonal antibodies specific for the Sp family transcription factors or normal rabbit IgG prior to the addition of ^{32}P-labeled wild-type probes: Sp1-1+2 (WT), (A); Sp1-3(WT), (B). Labeled probe alone (A and B, lanes 1); labeled probe incubated with nuclear extract in the absence of antibodies (A and B, lanes 2); in the presence of rabbit polyclonal antibodies: anti-Sp1 (A and B, lanes 4), anti-Sp2 (A and B, lanes 5), anti-Sp3 (A and B, lanes 6), anti-AP-2 (A, lane 7), normal rabbit IgG (A and B, lanes 3). Shifted bands are imarked with an asterisk. Results were analyzed by a phosphor imager.

doi:10.1371/journal.pone.0135141.g003

the faster migrating complex 2 (Fig 3A, lane 6). Both Sp1 and Sp2 specific antibodies reduced the complex formation with the Sp1-3 probe (Fig 3B, lanes 4 and 5), but a small amount of supershifted complex was also observed. In contrast, the control normal rabbit IgG (Fig 3A and 3B, lanes 3) had no effect. Taken together, supershift experiments showed that members of the Sp transcription factor family (Sp1, Sp2, Sp3) are involved in specific interactions with Sp1 binding sites at positions -641 and -635 within the h*FCGRT* promoter in the selected cell lines, whereas the Sp1 and Sp2 nuclear proteins specifically bind to the Sp1 sequence located at -313 in the h*FCGRT* promoter.

For the Ap1 site, one robust DNA-protein complex was identified in EMSAs using ^{32}P-labeled probe AP1 corresponding to the h*FCGRT* promoter sequence between nucleotides -284 and -254 containing the putative AP-1 binding site at position -276 (Fig 4A, lane 2). The specificity of the band complex was demonstrated in a competition assay with the unlabeled wild-type AP1 oligonucleotide (Fig 4A, lane 3), but not with its mutated version, in which the putative AP-1 binding site located at -276 was mutagenized (Fig 4A, lane 4). This complex was recognized by anti-c-Fos (Fig 4B, lane 3) and anti-c-Jun (Fig 4B, lane 4) antibodies, indicating that c-Jun and c-Fos proteins form heterodimers to bind the AP-1 site located at position -276 in the h*FCGRT* promoter.

The (CF1-1) oligonucleotide, corresponding to the h*FCGRT* sequence between nucleotides -593 and -565, was used to examine the binding of nuclear proteins to the CF1/YY1 motif located at -586 in the h*FCGRT* promoter. In the presence of the ^{32}P-labeled CF1-1 probe, a slow migrating complex was observed (Fig 5A, lane 2), which was partially competed by a 100-fold molar excess of unlabeled CF-1 oligonucleotide (Fig 5A, lane 3). Partial competition of the complex formed with the CF1-1 probe might be due to the sequence present in this

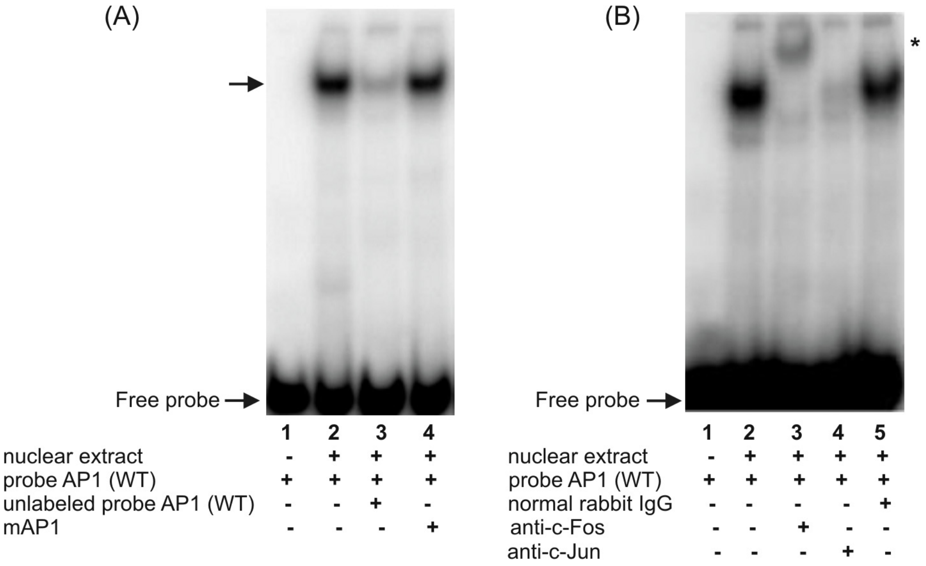

Fig 4. Characterization of the putative AP-1 binding site in the h*FCGRT* promoter by EMSA (A) and supershift analysis (B). EMSA and supershift experiments were performed using the AP1 probe corresponding to the h*FCGRT* promoter sequence between the nucleotides -284 and -254, which contained the putative AP-1 binding site at position -276. The arrow indicates the specific complex formed during incubation of the ^{32}P-labeled wild-type AP1 probe, AP1(WT), with the nuclear extract from differentiated THP-1 cells (A, lane 2). Competition experiments were performed in the presence of a 100-fold molar excess of unlabeled probe AP1(WT) (A, lane 3) or in the presence of its mutated version, in which the putative AP-1 binding site at position -276 was mutagenized–mAP1 (A, lane 4). Supershift experiments were performed by preincubating the nuclear extract with the rabbit polyclonal anti-c-Fos antibody (B, lane 3), anti-c-Jun antibody (B, lane 4), normal rabbit IgG (B, lane 5), prior to the addition of ^{32}P-labeled wild-type AP1 probe–AP1(WT). Labeled probe AP1(WT) alone (A and B, lanes 1); labeled probe AP1(WT) incubated with nuclear extract in the absence of antibodies (B, lane 2). Position of the shifted complex is marked by an asterisk. Results were analyzed by a phosphor imager.

doi:10.1371/journal.pone.0135141.g004

probe, which could interact nonspecifically with nuclear extract. A competitive effect was not observed when the unlabeled mCF1-1 oligonucleotide, containing a mutation in the CF1/YY1 motif was used (Fig 5A, lane 4). Complex formation was reduced by the polyclonal YY1 rabbit antibody (Fig 5A1, lane 3), but not by the normal rabbit IgG (Fig 5A1, lane 4) or the polyclonal rabbit anti-Sp1 antibody (Fig 5A1, lane 5). As shown in Fig 5B, the interaction of the ^{32}P-labeled CF1-2 oligonucleotide probe (containing the potential CF1/YY1 transcription regulatory motif at position -357) with nuclear extracts resulted in two specific protein-DNA complexes (Fig 5B, lane 2). The specificity of binding was demonstrated by a competition assay. Both complexes were effectively competed by an excess of the unlabeled CF1-2 oligonucleotide (Fig 5B, lane 3), but not by the mutant CF1-2 oligonucleotide (Fig 5B, lane 4). These specific complexes were supershifted by polyclonal rabbit antibody to YY1 (Fig 5B1, lane 3). In contrast, the normal rabbit IgG had no effect (Fig 5B, lane 4). Thus, the weak second complex that formed with the CF1-2 probe might represent more phosphorylated or glycosylated form of the YY1 protein than the first complex. The results indicate that the YY1 family of transcription factors is involved in the specific interaction with the CF1/YY1 transcriptional regulatory motif located at -586 and -357 within the h*FCGRT* promoter. The lower mobility of the DNA-protein complex formed with the CF1-1 oligonucleotide (Fig 5A, lane 2), compared with migration in polyacrylamide gel electrophoresis of a complex formed with the CF1-2 probe (Fig 5B, lane 2), suggests that different isoforms of YY1 can bind to these CF1/YY1 elements. It is known that there are eight different protein isoforms of human YY1 encoded by eight

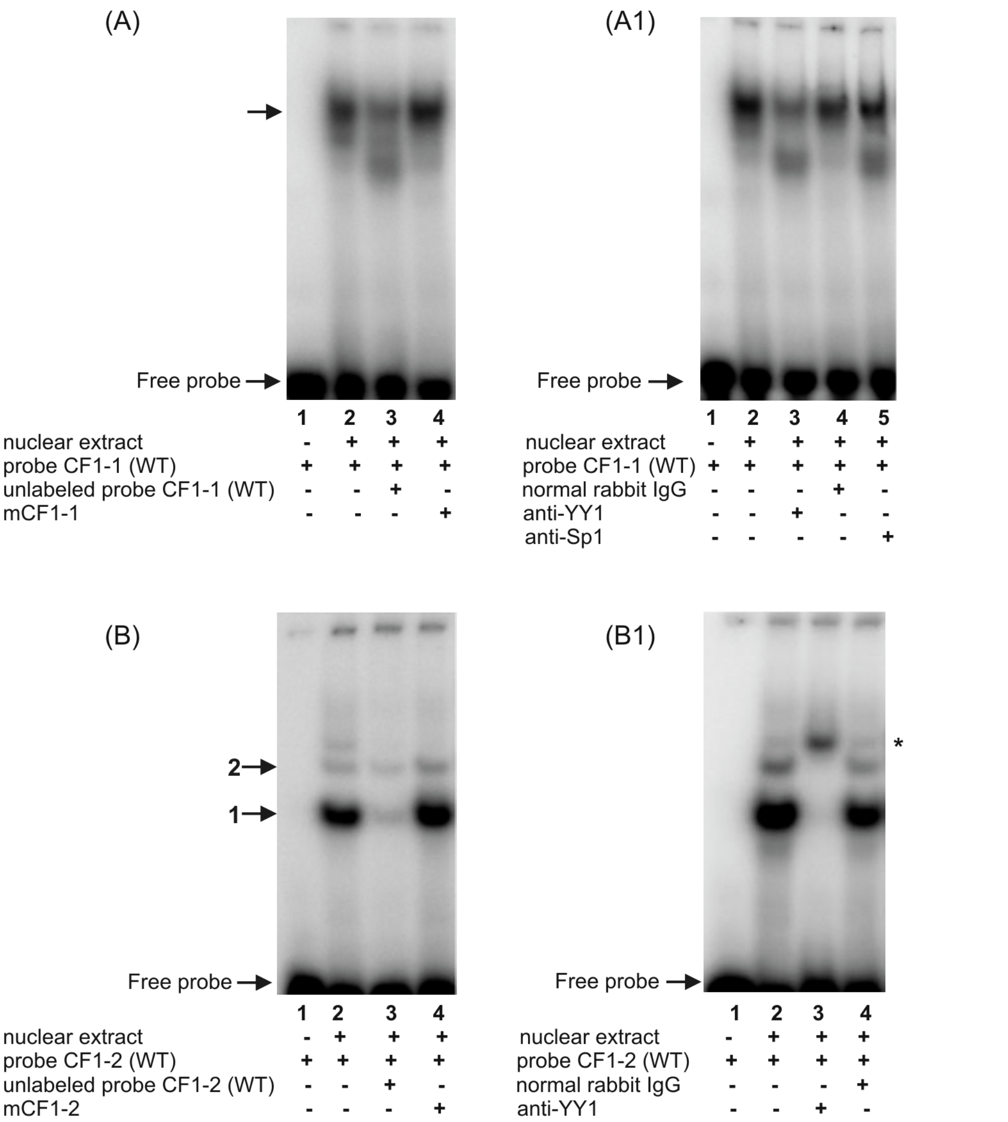

Fig 5. Characterization of two potential CF1/YY1 regulatory motifs in the -660/-233 fragment of the h*FCGRT* promoter by EMSA (A, B), and supershift analysis (A1, B1). EMSA and supershift experiments were carried out using the following probes: CF1-1 **–** double-stranded oligonucleotide representing the h*FCGRT* promoter sequence between the nucleotides -593 to -565, containing the putative CF1/YY1 binding site at position -586 (A, A1); CF1-2 – ds-oligonucleotide corresponding to the -362/-337 sequence of the h*FCGRT* promoter containing the potential CF1/YY1 transcriptional regulatory motif at position -357 (B, B1). In EMSA, ^{32}P-labeled wild-type probes were incubated with nuclear extract in the absence of competitor (A and B, lanes 2) or in the presence of a 100-fold molar excess of competitor: CF1-1(WT)–unlabeled wild type probe CF1-1 (A, lane 3), CF1-2 (WT)–unlabeled wild type probe CF1-2 (B, lane 3), mCF1-1 – probe CF1-1 with mutation in the CF1 element at position -586 (A, lane 4), mCF1-2 – probe CF1-2 containing mutation in the CF1 site at position -357 (B, lane 4). Supershift experiments were performed by preincubating the nuclear extract with the anti-YY1 rabbit polyclonal antibody (A1 and B1, lanes 3), anti-Sp1 antibody (A1, lane 5), normal rabbit IgG (A1 and B1, lanes 4), prior to the addition of ^{32}P-labeled wild-type probe: CF1-1(WT) (A1); CF1-2(WT) (B1). Labeled probes incubated with nuclear extract in the absence of antibodies (A1 and B1, lanes 2). Labeled probe CF1-1(WT) alone (A and A1, lanes 1); labeled probe CF1-2 (WT) alone (B and B1, lanes 1). Arrows indicate the specific DNA-protein complexes and the asterisk indicates the supershift complex. Results were analyzed by a phosphor imager.

doi:10.1371/journal.pone.0135141.g005

different transcripts generated by alternative splicing [33]. However, it cannot be excluded that YY1 forms a heterodimer with a transcription factor from different family and this

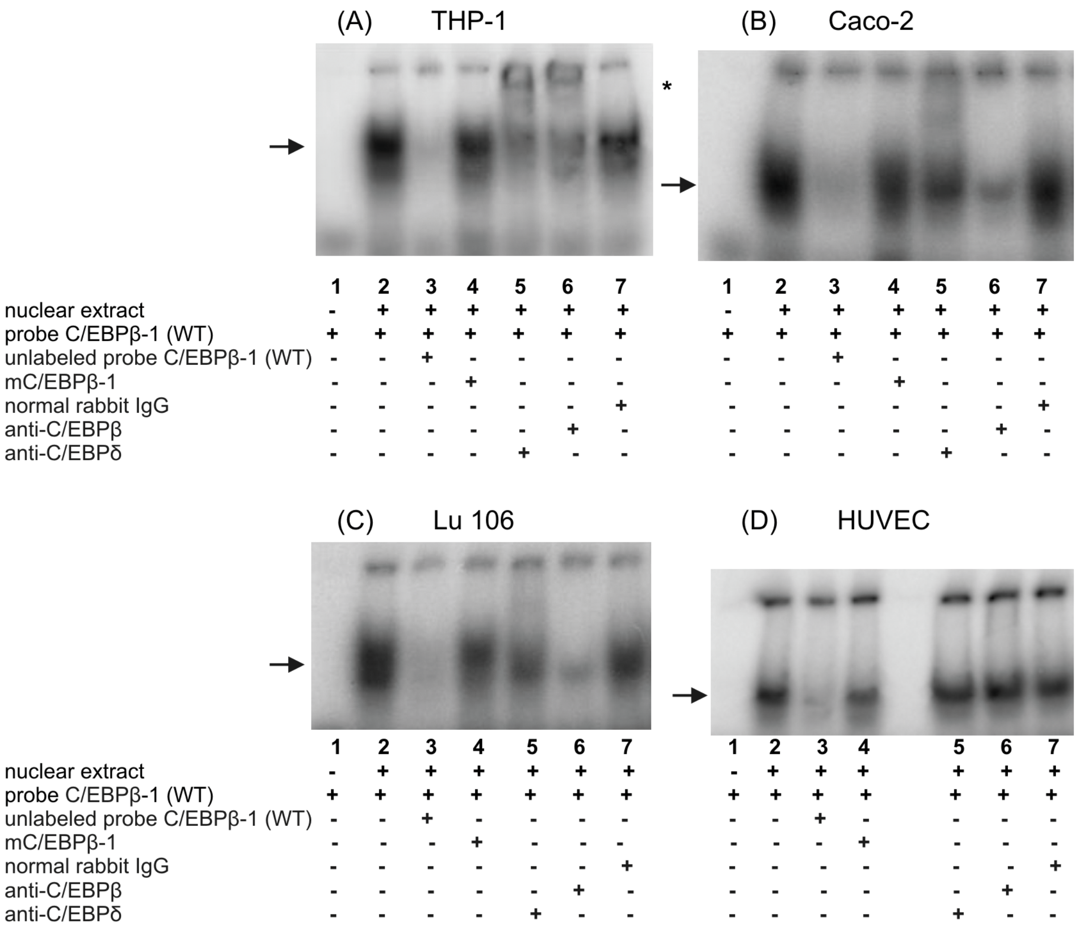

Fig 6. Characterization of the putative C/EBPβ binding site at position -497 within the -660/-233 fragment of the h*FCGRT* promoter. EMSA and supershift experiments were performed using C/EBPβ-1 probe corresponding to the h*FCGRT* promoter sequence between the nucleotides -499 and -474, which contained the putative C/EBPβ binding site at position -497. Arrows indicate the specific DNA-protein complex formed during incubation of the ^{32}P-labeled wild-type C/EBPβ-1 probe, C/EBPβ-1(WT), with the nuclear extract from differentiated THP-1 cells (A, lane 2), Caco-2 (B, lane 2), Lu 106 (C, lane 2), HUVEC (D, lane 2). Competition experiments were carried out in the presence of a 100-fold molar excess of the unlabeled C/EBPβ-1 (WT) probe, (A-D, lanes 3), or in the presence of its mutated version, mC/EBPβ-1, in which the putative C/EBPβ binding site at position -497 was mutagenized (A-D, lanes 4). Supershift experiments were performed by preincubating the nuclear extracts with the anti-C/EBPδ polyclonal rabbit antibody (A-D, lanes 5); anti-C/EBPβ (A-D, lanes 6); normal rabbit IgG, (A-D, lanes 7) before addition of the ^{32}P-labeled wild-type C/EBPβ-1 probe. The ^{32}P-labeled wild-type C/EBPβ-1 probe alone (A-D, lanes 1). Shifted complexes are marked with an asterisk. Results were analyzed by a phosphor imager.

doi:10.1371/journal.pone.0135141.g006

heterodimeric complex binds to the CF1/YY1 motif at -586. There is evidence that YY1can associate with the Sp1 transcription factor [34,35]. However, in this study, anti-Sp1 antibody did not recognize the Sp1 factor in the DNA-protein complex formed with a probe containing the CF1/YY1 binding site at -586 (Fig 5A1, lane 5).

Two consensus C/EBPβ binding sequences for the transcription factor C/EBPβ were identified at positions -497 and -233 within the-660/-233 fragment of the h*FCGRT* promoter. The C/EBPβ transcription factor is expressed at a low level in normal cells, but can be drastically and rapidly induced in many cells by cytokines and mitogens such as LPS or PMA, indicating that C/EBPβ may be involved in the transcriptional regulation of genes in inflammatory situations. Expression of C/EBPβ was shown to be increased during monocyte differentiation [36,37]. The binding activity of these potential C/EBPβ sites in nuclear extracts from differentiated THP-1 cells as well as from stimulated epithelial and endothelial cells was examined by electrophoretic mobility shift assay.

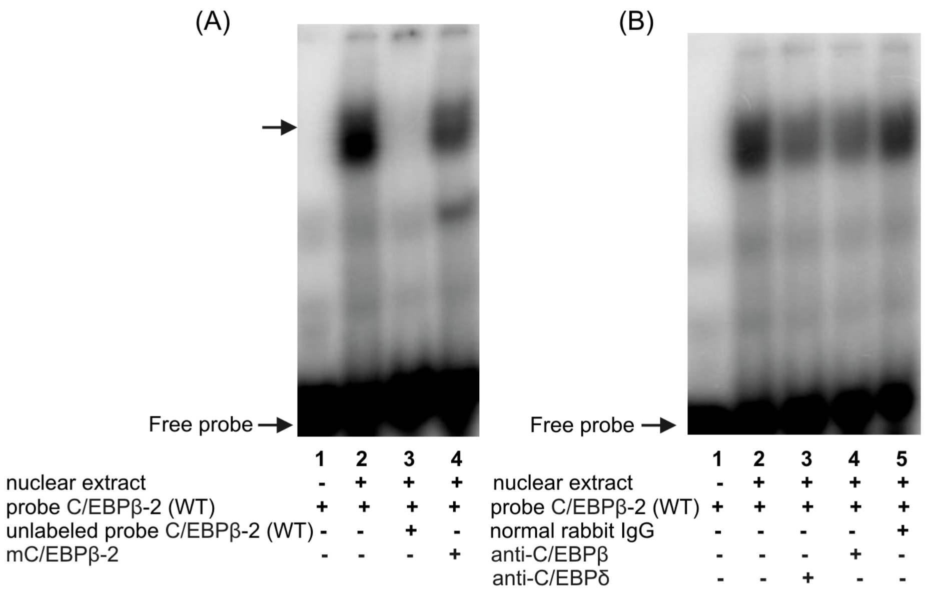

Fig 7. Characterization of the putative C/EBPβ binding site at position -233 withinthe h*FCGRT* promoter by EMSA (A) and supershift analysis (B). EMSA and supershift experiments were performed using the C/EBPβ-2 probe–ds-oligonucleotide representing the -240/-211 sequence of the h*FCGRT* promoter, which contains the potential C/EBPβ transcriptional regulatory motif at position -233. Nuclear extracts were isolated from differentiated THP-1 cells treated with LPS for 1 h. The arrow indicates the specific DNA-protein complex formed during incubation of the ^{32}P-labeled wild-type C/EBPβ-2 probe–C/EBPβ-2(WT), with nuclear extract (A, lane 2). Competition experiments were carried out in the presence of a 100-fold molar excess of the unlabeled wild-type C/EBPβ-2 probe (A, lane 3), or in the presence of the unlabeled C/EBPβ-2 probe (mC/EBPβ-2) containing mutation in the putative C/EBPβ binding site at position -233 (A, lane 4). Supershift experiments were performed by preincubating the nuclear extract with the polyclonal rabbit anti-C/EBPδ antibody (B, lane 3), anti-C/EBP (B, lane 4), normal rabbit IgG (B, lane 5), before addition of the ^{32}P-labeled wild-type C/EBPβ-2 probe. The ^{32}P-labeled wild-type C/EBPβ-2 probe incubated with nuclear extract in the absence of antibodies (B, lane 2); labeled probe C/EBPβ-2(WT) alone (A and B, lanes 1). Results were analyzed by a phosphor imager.

doi:10.1371/journal.pone.0135141.g007

Incubation of nuclear extracts from the PMA-differentiated THP-1 cells with the radiolabeled C/EBPβ-1 probe corresponding to the h*FCGRT* promoter sequence -499/-474, which contains the putative C/EBPβ binding site located at -497, generated a single DNA-protein complex (Fig 6A, lane 2). This complex was specific, because a 100-fold molar excess of the unlabeled wild-type C/EBPβ-1 oligonucleotide eliminated the complex formation (Fig 6A, lane 3), but not the mutant mC/EBPβ-1 oligonucleotide with mutation in the C/EBPβ motif at -497 (Fig 6A, lane 4). Supershift experiments showed that the specific DNA-protein complex was reduced and supershifted by the anti-C/EBPβ antibody (Fig 6A, lane 6) and antibody against C/EBPδ (Fig 6A, lane 5). In contrast, the control normal rabbit IgG (Fig 6A, lane 7) exerted no effect. These results indicate that the heterodimeric transcription factors C/EBPβ and C/EBPδ of the C/EBP family participate in specific interactions with the C/EBPβ binding site at position -497 within the h*FCGRT* promoter

Specific complexes were also formed between the C/EBPβ-1 oligonucleotide probe and the nuclear extracts isolated from epithelial (Fig 6B and 6C) and endothelial cells (Fig 6D) after 2–6 h LPS or PMA treatment. However, in epithelial cells, C/EBPβ homodimers, and in part C/EBPδ dimers, interacted with the C/EBPβ site at -497 in the h*FCGRT* promoter, since the DNA-protein complexes formed were reduced predominantly by the anti-C/EBPδ antibody (Fig 6B and 6C, lanes 6) and partially by anti-C/EBPδ (Fig 6B and 6C, lanes 5). In contrast, antibodies against C/EBPβ or C/EBPδ had no effect on the specific complexes formed between

the C/EBPβ-1 oligonucleotide and nuclear extracts from LPS- or PMA- stimulated endothelial cells (Fig 6D, lanes 5 and 6). The inability of anti-C/EBPβ or anti-C/EBPδ antibody to supershift these complexes or abrogate their formation may be explained by recognition incompetence due to the phosphorylation state of C/EBPβ or C/EBPδ or conformational changes in their structure as a result of association with other nuclear proteins. The binding of nuclear proteins to the putative C/EBPβ binding site located at -233 in the h*FCGRT* promoter was observed only with the nuclear extract from THP-1 cells after treatment with PMA and LPS. A single DNA-protein band was revealed when the double-stranded C/EBPβ-2 oligonucleotide, representing the h*FCGRT* promoter sequence -240/-211, containing the C/EBPβ motif at -233, was used as a probe (Fig 7A, lane 2). This complex was abrogated when competition assay was carried out using the wild-type unlabeled C/EBPβ-2 oligonucleotide (Fig 7A, lane 3). Formation of this complex was not affected when the mC/EBPβ-2 oligonucleotide, which contains point mutations in the C/EBPβ binding site (Fig 7A, lane 4) was used as a competitor. These results suggested that the binding of nuclear proteins to the -240/-211 sequence of the h*FCGRT* promoter occurred specifically at the C/EBPβ site. Supershift analysis using antibodies against C/EBPδ (Fig 7B, lane 3) and C/EBPβ (Fig 7 B, lane 4) indicated that this DNA-protein complex was formed by the interaction of the C/EBPβ site at -233 bp in the h*FCGRT* promoter with C/EBPβ and C/EBPδ transcription factors.

Effect of specific mutations in the h*FCGRT* promoter on its activity

To examine whether the identified binding sites for transcription factors Sp1/Sp2/Sp3, CF1/YY1, AP1 (c-Fos/c-Jun) and C/EBPβ/CEBPδ function as regulators of the h*FCGRT* promoter activity, several mutant reporter constructs were generated, as described in Materials and Methods, and analyzed in transient transfection experiments in Caco-2, Lu 106, HSkMEC, and PMA-differentiated THP-1 cell lines. HUVEC cells were also initially used for transfection experiments, but their transfection efficiency was found to be very low, which caused transient transfection tests difficult to interpret for any of the reporter plasmids studied. Therefore, the HSkMEC endothelial cell line, which showed a significantly higher transfection efficiency, was used in transient transfection experiments. Table 2 presents the oligonucleotides used for site-directed mutagenesis. These mutations were identical to the mutations tested in the EMSA assays, and, as judged by competition experiments, they did not block the formation of DNA-protein complexes (Figs 2–7 and S1–S4 Figs)

To investigate the function of individual Sp1 sites, each Sp1 site alone or in several combinations was mutated in the pGl3-711(WT) promoter construct. The activities of mutant promoters were compared to that of the wild-type promoter construct. As shown in Fig 8B, the pGL3-711(WT) wild-type construct increased the luciferase activity 10-fold in Caco-2, THP-1, HSkMEC cell lines, and 20-fold in Lu 106 cells compared with the promoterless pGL3-basic vector. Mutation of the Sp1 binding site at -641 [pGl3-711(mSp1-1) construct] and -635 [pGl3-711(mSp1-2) construct] as well as the double Sp1 mutant [pGL3-711(mSp1-1+2)] reduced the h*FCGRT* promoter activity to about 50% of the wild-type promoter activity in Caco-2, HSkMEC and THP-1 cell lines, and 56–66% in Lu 106 cells, suggesting that the two sites are involved in transcription of the human *FCGRT* gene in an interdependent manner. Mutation of the Sp1 site at -313 [pGl3-711(mSp1-3) construct] had a dramatic effect on the promoter activity in all the selected cells, as there was an 85–89% decrease observed in the luciferase activity. When the Sp1 sites at positions -641, -635, and -313 were mutated simultaneously [pGL3-711(mSp1-1+2+3) construct], luciferase expression driven by this mutant construct was comparable to that of the wild-type pGL3-711 (WT) in Lu 106 cells, while in Caco-2, THP-1 and HSkMEC cells it was reduced only to 55–60% of the wild-type promoter

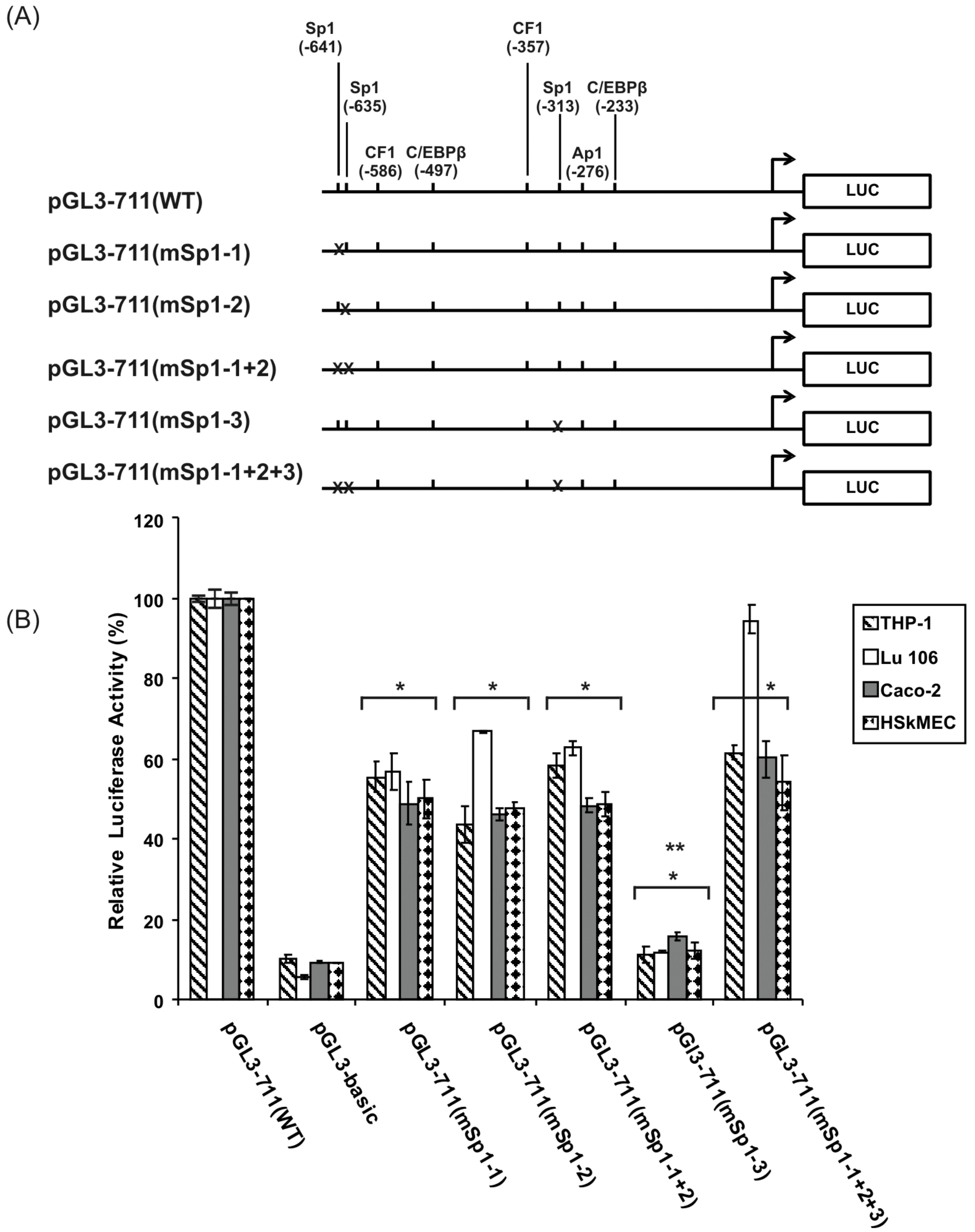

Fig 8. Role of Sp1 binding sites in the promoter activity of the human *FCGRT* gene. (A) Schematic diagram representing the wild-type promoter construct and the mutant promoter constructs. Mutations are marked with crosses. (B) Summary of the results of luciferase activity. Mutant promoter constructs were generated from the wild-type pGL3-711(WT) subjected to site-directed mutagenesis by the QuikChange Lightning Site-directed Mutagenesis Kit, as described in Materials and Methods. Mutant promoter constructs pGL3-711(mSp1-1), pGL3-711(mSp1-2), pGL3-711(mSp1-1+2), pGL3-711(mSp1-3) and pGL3-711(mSp1-1 +2+3) contain mutations in the Sp1sequence at positions -641, -635, -641 plus -635, -313, -641 plus -635 plus -313, respectively. Using TransIT-2020 transfection reagent, pGL3-basic vector, wild-type pGL3-711 (WT) construct and site-directed mutant constructs were transiently cotransfected with the phRG-B plasmid (internal control) into THP-1, Lu 106, Caco-2, and HSkMEC cell lines. Twenty-four hours after transfection, the cells were harvested and luciferase activity was measured. The transfected THP-1 cells were exposed to PMA (100 ng/ml) for 4 hours prior to harvesting. Transfection efficiency was normalized to the *Renilla* luciferase activity derived from the phRG-B plasmid and expressed as relative luciferase activity. The data shown are means ± SD of three to six independent experiments performed in duplicate for each construct. The transcriptional activity of the mutant constructs and pGL3-basic is plotted as a percentage of the normalized activity of the wild-type pGL3-711(WT) construct, which is defined as 100%. The differences

between the wild-type construct and mutant constructs were statistically significant; *P 0.01 vs. pGL3-711 (WT) construct; **P 0.01 compared with pGL3-711(mSp1-1), pGL3-711(mSp1-2), pGL3-711(mSp1-1+2) and pGL3-711(mSp1-1+2+3).

doi:10.1371/journal.pone.0135141.g008

activity, essentially abating the positive effect of the Sp1 site at -313. These results indicated that the Sp1 sites located at positions -641, -635, and -313 acted as positive regulators of the h*FCGRT* promoter activity. The Sp1 motif at -313 may be the most important regulatory sequence for transcription of the human *FCGRT* gene in all model cells, because mutation of this site had a stronger effect on promoter activity than did mutations in the Sp1 binding sites at -641 and -635. The data also suggested that the partly overlapping Sp1 sites at positions -641 and -635 might suppress the positive effect of the Sp1 sequence at-313 on the human *FCGRT* gene transcription.

The pGL3-711(mAP1) construct carrying mutation in the AP-1 motif demonstrated drastic reduction in the ability to drive the reporter activity (Fig 9B), suggesting that the AP-1 site plays an essential role in the activation of the h*FCGRT* promoter in Caco-2, Lu 106, HSkMEC and THP-1 cells. Mutation of the AP-1 binding site at position -276 caused a decrease in the activity of the h*FCGRT* promoter in Caco-2 and HSkMEC cells to a level that was not significantly different from that of the promoterless luciferase plasmid pGL3-basic, while in Lu106 and THP-1 cells the activity of the promoter was decreased to 23% and 29%, respectively, compared to the wild-type promoter activity.

Functional analysis of the CF1/YY1 binding sites within the h*FCGRT* promoter is presented in Fig 10.

Mutation of the CF1/YY1 binding site at position -586 [pGL3-711(mCF1-1) construct] reduced the promoter activity by about 60% in Caco-2 and Lu 106 cells, and by 47% in HSkMEC cells. Mutation of the CF1/YY1 motif at position -357 [pGL3-711(mCF1-2 construct] reduced the initial h*FCGRT* promoter activity to about half in these cell lines. The CF1 double mutant [pGL3-711(mCF1-1+2) construct] did not cause a more drastic reduction in the promoter activity, suggesting the presence of a potential interaction between nuclear factors that bind to these *cis* elements. In contrast, mutation of the CF1/YY1 site at -586 resulted in a significant decrease in reporter activity in the THP-1 cell line, comparable to the activity of pGL3-basic, indicating that this CF1/YY1 site functions as a critical positive regulatory element of the h*FCGRT* promoter in THP-1 cells. On the other hand, mutation of the CF1/YY1 site at -357 and the CF1double mutant caused a very slight decrease of the promoter activity in this cell line, suggesting that the strong activation of the h*FCGRT* promoter in THP1 cells by the CF1 site at -586 is neutralized by the repressive effect of the CF1/YY1 motif at position -357.

The activity of the h*FCGRT* promoter was increased 2-fold in Caco-2, Lu 106 and HSkMEC cell lines stimulated by PMA (Fig 11B) and LPS (Fig 11C) in comparison with unstimulated cells. When the C/EBPβ site at -497 was mutated [pGL3-711(mC/EBPβ-1) construct], promoter activity was reduced by almost 50%, reaching a level comparable to that driven by the wild-type pGL3-711(WT) construct in untreated cells.

As shown in Fig 12, treatment of THP-1 cells with PMA alone or PMA + LPS had a similar effect on the human *FCGRT* gene transcription. The activity of the h*FCGRT* promoter was was only slightly reduced by the mutation in the C/EBPβ site at -497 [pGL3-711(mC/EBPβ-1) construct], with the C/EBPβ site at -233 left intact, or in the opposite situation when the mutation was introduced in the C/EBPβ site at -233 [pGL3-711(mC/EBPβ-2) construct] and the C/EBPβ site at -497 was left unchanged. Mutations in both C/EBPβ motifs [pGL3-711(mC/EBPβ-1+2) construct] resulted in a decreased activity of the h*FCGRT* promoter by almost 50% in THP-1

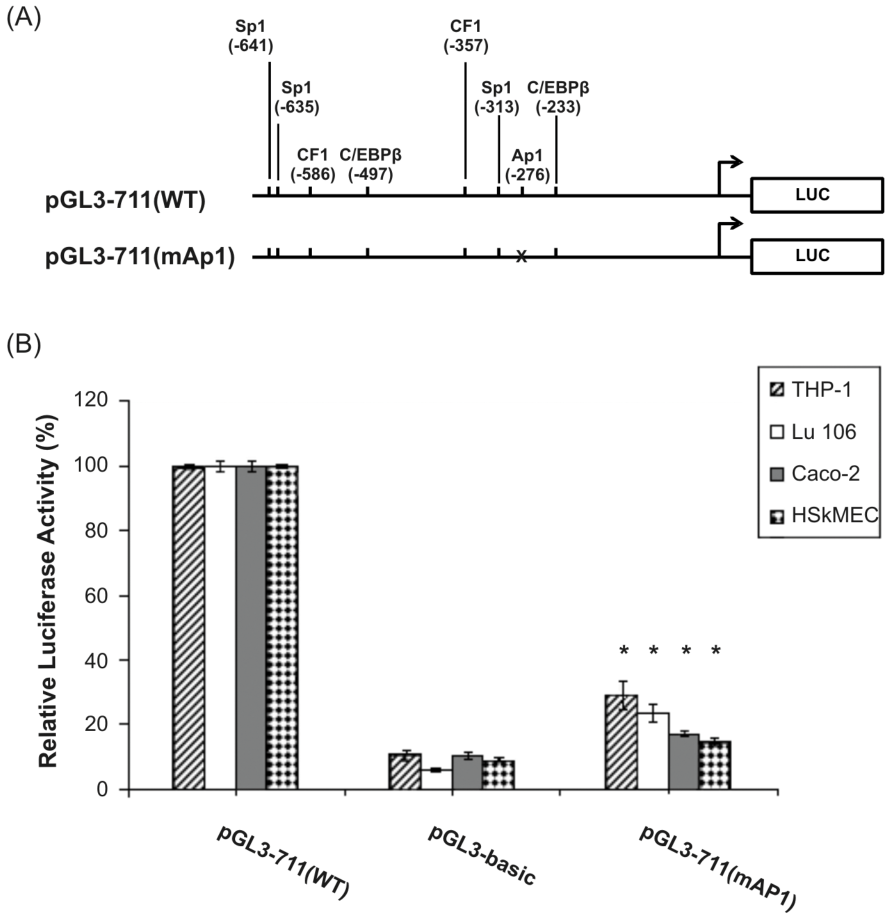

Fig 9. Effect of the AP-1 binding motif on the transcriptional activity of the h*FCGRT* promoter. (A) Schematic diagram representing wild-type and mutant promoter constructs. Mutations are marked with crosses. (B) Summarized results of luciferase activity tests. The pGL3-711(mAP1) mutant construct was prepared as described in Materials and Methods. This construct is a derivative of the pGl3-711(WT) wild-type construct containing a mutation in the AP-1 binding site at position -276. Transfection and normalization were performed as described in the legend to Fig 8. Values are expressed as means ± SD of three to six independent experiments performed in duplicate. The promoter activity of mutant constructs and pGL3-basic is represented as a percentage of the normalized activity of the wild-type pGL3-711(WT) construct, which is defined as 100%. Significant repression of transcription compared with the wild-type construct, *P 0.01 versus pGL3-711(WT).

doi:10.1371/journal.pone.0135141.g009

treated with PMA+LPS (Fig 12C), and 40% in cells treated with PMA (Fig 12B) compared to the activity of the wild-type promoter.

Taken together, the data suggest that the C/EBPβ binding site at -497 in the h*FCGRT* promoter in Caco-2, Lu 106, and HSkMEC cells, and the C/EBPβ binding site located at positions -497 and -233 within the h*FCGRT* promoter in THP-1 cells functioned as positive regulators of the human *FCGRT* gene transcription under stimulated conditions.

Fig 13 shows the regulatory elements identified in the -660/-233 fragment of the h*FCGRT* promoter that participate in transcriptional regulation of the human *FCGRT* gene.

Discussion

A substantial body of research exists on the structure, function and physiological significance of the hFcRn receptor. However, the mechanism by which the expression of the human

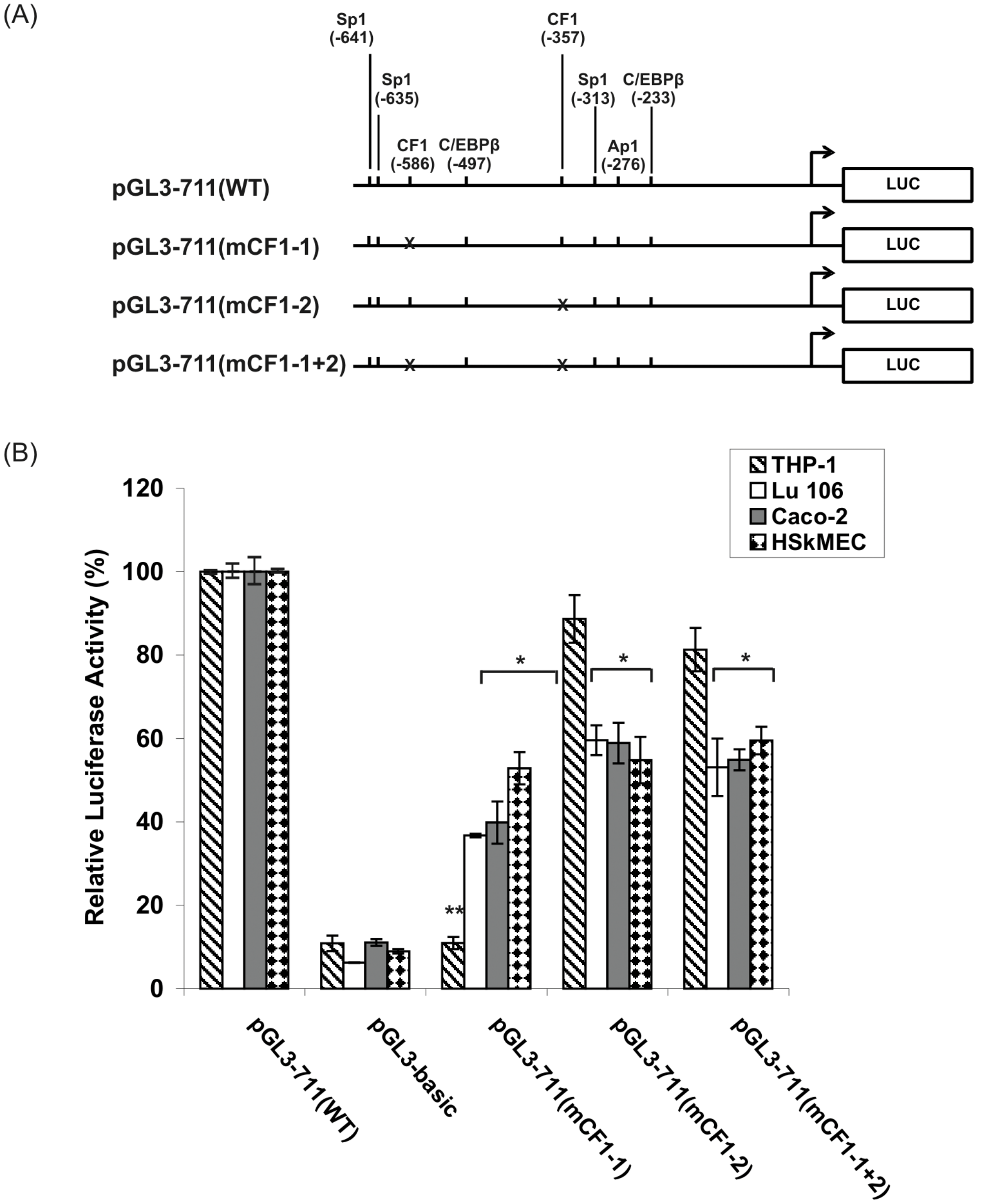

Fig 10. Functional analysis of the CF1 binding sites within the h*FCGRT* promoter. (A) Schematic diagram representing the wild-type promoter construct and the mutant promoter constructs. Mutations are marked with crosses. (B) Summarized results of luciferase activity tests. The mutant constructs pGL3-711 (mCF1-1), pGL3-711(mCF1-2), and pGL3-711(mCF1-1+2) are derivatives of the wild-type pGL3-711(WT) with mutation in the CF1 site at positions-586, -357, -586 plus -357, respectively. Transfection and normalization were performed as described in the legend to Fig 8. All results represent means ± SD of three to six independent experiments performed in duplicate and plotted as a percentage of the normalized activity of the wild-type pGL3-711(WT) construct, which is defined as 100%. The promoter activity of the labeled construct is significantly decreased compared to the wild-type construct; *P 0.01 vs. pGL3-711(WT); **P 0.01 compared with pGL3-711(mCF1-2) and pGL3-711(mCF1-1+2) in THP-1.

doi:10.1371/journal.pone.0135141.g010

FCGRT gene (encoding human FcRn α chain) is regulated remains unknown. Previous publications presenting the exon-intron structure and sequence of the human *FCGRT* gene [26], as well as partial characterization of the promoter region of this gene [27], served as a basis for

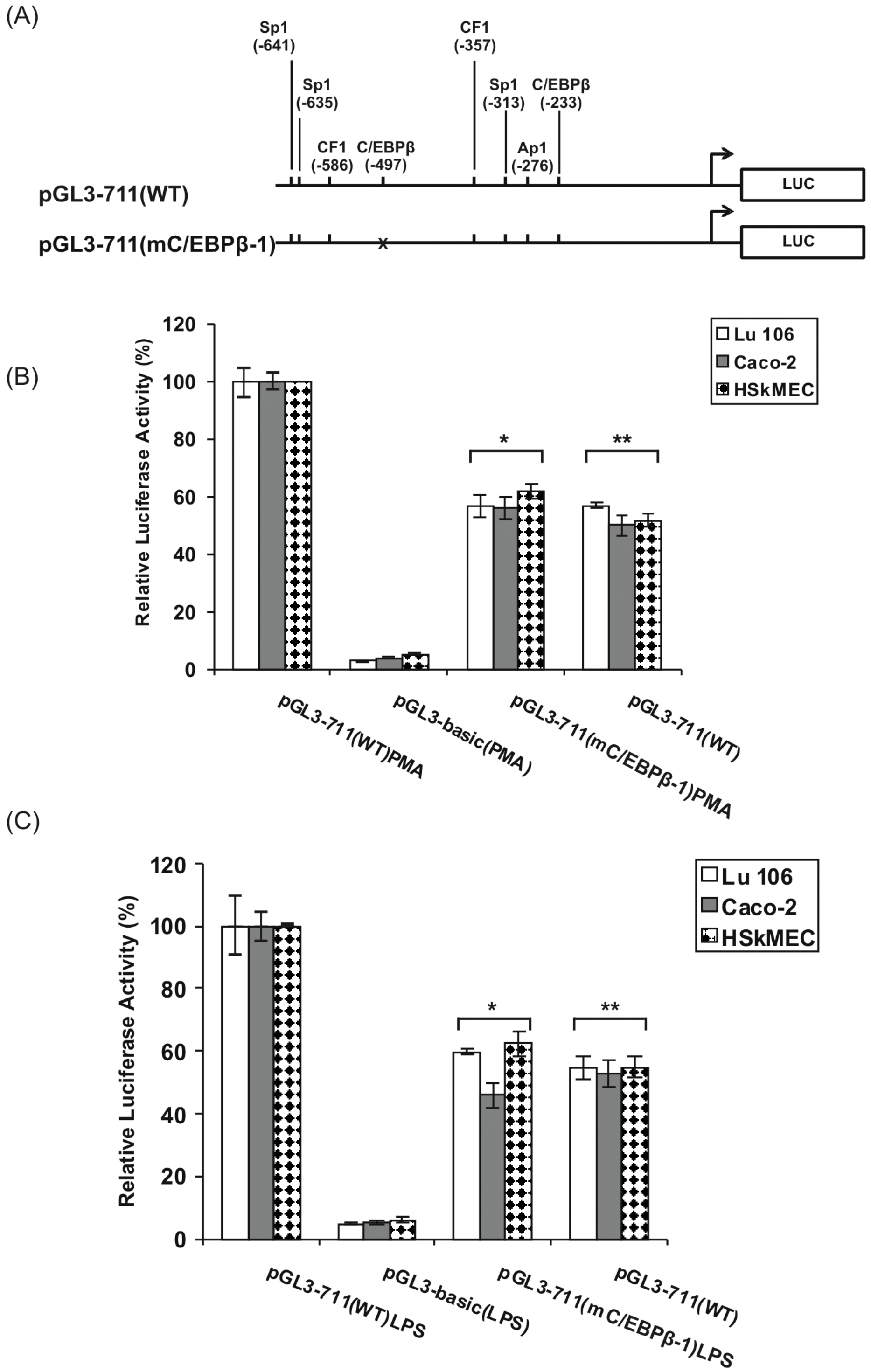

Fig 11. Effect of mutation in the C/EBPβ binding site on the h*FCGRT* promoter activity in Lu 106, Caco-2, and HSkMEC cell lines. (A) Schematic diagram representing wild-type and mutant promoter constructs. Mutations are marked with crosses. (B and C) Summarized results of luciferase activity tests. The pGL3-711(mC/EBPβ-1) mutant construct, containing a mutation in the C/EBPβ sequence at position -497, was prepared as described in Materials and Methods. Transfection and normalization were performed as described in the legend to Fig 8. Twenty-four hours after transfection, the cells were exposed to PMA(100 ng/ml) (B) and LPS (5 μg/ml) (C) for 6 hours and harvested for luciferase assays. The cells after transfection with the wild-type pGL3-711(WT) construct were cultured for 6 hours in media without LPS and PMA prior to

harvesting (control). The promoter activity of the mutant constructs and pGL3-basic plasmid is represented as a percentage of the normalized activity of the wild-type promoter construct in the presence of PMA (pGL3-711(WT)PMA construct) or LPS (pGL3-711(WT)LPS construct), which is defined as 100%. Data shown are means ± SD of three to six independent experiments performed in duplicate. Significant reduction in the promoter activity of the mutant construct compared to wild-type construct, *P 0.01 vs. pGL3-711(WT)PMA and pGL3-711(WT)LPS. **–denotes a significant difference (**P 0.01) between the promoter activity of the wild-type construct in stimulated (PMA or LPS) and unstimulated cells.

doi:10.1371/journal.pone.0135141.g011

studying the regulation of the human *FCGRT* expression at the level of transcription. Deletion of the 434 bp segment of the h*FCGRT* promoter revealed that the sequence from nt -660 to -233 is essential for the basal promoter function [27]. Removal of this part of the h*FCGRT* promoter abolished almost all of the promoter activity. The potential binding sites for the Sp family as well as YY1, AP-1, and C/EBPβ factors were predicted within this region based on computer analysis. The aim of this study was to examine whether these potential regulatory elements are involved in h*FCGRT* promoter activation in human epithelial, endothelial, and differentiated THP-1 cell lines, and to identify transcription factors that specifically bind to these motifs.

The results obtained from site-directed mutagenesis in transient transfection experiments (Figs 8–10) and two-way ANOVA analysis of these data indicated that the Sp1 sites at positions -641, -635, and -313, the CF1/YY1 elements at positions -586 and -357, and the AP-1 motif at -276 within the -660/-233 fragment of the h*FCGRT* promoter, act interdependently and functionally cooperate in the regulation of constitutive transcription of the human *FCGRT* gene in epithelial, endothelial, and differentiated THP-1 cells. However, their individual contribution to the promoter activity is not equivalent. The Sp1-binding site at -313 and the AP-1 site at -276 are critical for the activity of the h*FCGRT* promoter in epithelial and endothelial cells. Moreover, the CF1/YY1 site at -586 in differentiated THP-1 cells plays an essential role in transcriptional activity of the h*FCGRT*. Mutation of the Sp1 site at -313, the AP-1 motif at -276 or the CF1/YY1 site at -586 (in differentiated THP-1 cells) results in a dramatic loss of promoter activity, strongly suggesting cooperative interactions between nuclear factors that bind to these *cis*-elements, so that the disruption of binding of any factor leads to a strong destabilization of the transcriptional machinery. It seems likely that this is an important step required to initiate the transcription machinery. These interactions may be necessary to open the nucleosomal structure facilitating transcription. It is possible that they play a role in the recruitment of transcription initiation complex onto the core promoter. This possibility cannot be excluded, considering the fact that the h*FCGRT* promoter does not contain a typical TATA sequence and Inr element. Reports concerning interactions between Sp1 and TBP or TAF 110 [38], the ability of YY1 to directly recruit TFIIB and Pol II, as well as the ability of Sp1 and YY1 factors to induce DNA bending [39] also support this hypothesis. The other Sp1 and CF1 sites in the h*FCGRT* promoter, mutation in which caused only a modest decrease in the promoter activity, are also likely to be operative in the regulation of the human *FCGRT* gene. These sites may have an effect on the function of the key transcriptional activators *via* recruitment of the transcriptional coactivators or/and by a competive mechanism. As it is shown in Fig 8B, mutation of the Sp1 motif at -313 reduced promoter activity by ~90%. When all the Sp1 sites (at positions -641, -635, and -313) were mutated [pGl3-711(mSp1-1+2+3) construct], luciferase expression driven by this triple mutant was higher than by the construct carrying the Sp1 mutation at -313. In the THP-1 cell line, luciferase activity of the pGL3-711(mCF1-1) construct, containing a mutation in the CF1/YY1 site at -586, was not significantly different from that of the promoterless luciferase plasmid, while the double mutant [pGL3-711(mCF1-1+2) construct] demonstrated similar activity to the wild type (Fig 10B). These results suggest that it is possible that the Sp1 site at

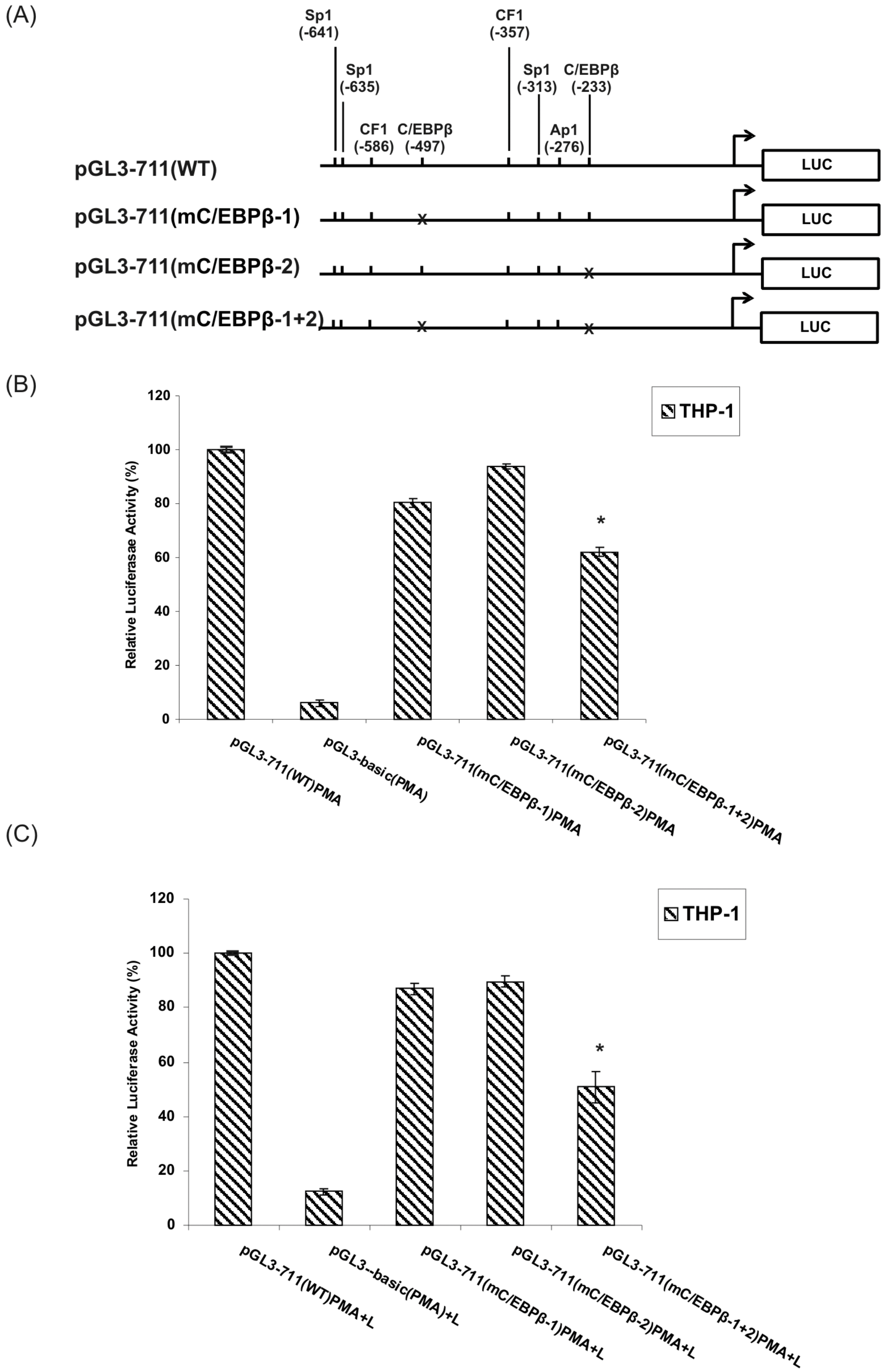

Fig 12. Effect of the mutation of C/EBPβ motifs on the h*FCGRT* promoter activity in THP-1 cells. (A) Schematic diagram representing wild-type and the mutant promoter constructs. Mutations are marked with crosses. (B and C) Summary results of luciferase activity tests. Mutant constructs pGL3-711(mC/EBPβ-1), pGL3-711(mC/EBPβ-2), and pGL3-711(mC/EBPβ-1+2) contain a mutation in the C/EBPβ site at positions -497, -233, -497 plus -233, respectively. These mutant constructs were prepared as described in Materials

and Methods. Transfection and normalization were performed as described in the legend to Fig 8. Following transfection, cells were incubated in complete medium with PMA (100 ng/ml) for 4 hours (B). The cells treated with PMA were cultured in the presence of LPS (5 μg/ml) for further 11 h (C). Values are expressed as means ± SD of four independent experiments performed in duplicate. The promoter activity of the mutant constructs and pGL3-basic plasmid is represented as a percentage of the normalized activity of the wild-type promoter construct in the presence of PMA (pGL3-711(WT)PMA construct) or PMA + LPS (pGL3-711(WT) PMA+L construct), which is defined as 100%. Mutation of both C/EBPβ binding sites led to a significant reduction in promoter activity, *P 0.05 vs. pGL3-711(WT)PMA or pGL3-711(WT)PMA+L.

doi:10.1371/journal.pone.0135141.g012

-641 and -635 competes with the Sp1 site at -313 for the Sp1 factor, while in differentiated THP-1 cells, the CF1/YY1 site at -357 competes with the CF1/YY1 site at -586 for the YY1 factor, and thus, the increased activation induced by the Sp1 element at -313 or CF1 motif at -586 can be balanced.

Recently, it was shown that TNF-α and IL-1 β up-regulated the transcription of the human *FCGRT* gene in THP-1 and Caco-2 cell lines through binding NF-κB transcription factors to NF-κB specific binding sites located in introns 2 and 4 [40]. However, INF-γ down-regulated the expression of this gene *via* JAK-STAT signaling [41]. It was also demonstrated that the NF-κB transcription factor was involved in the regulation of bovine *FCGRT* expression under stimulated conditions [42]. An interesting observation in this study was that the C/EBPβ binding site at -497 in the h*FCGRT* promoter in epithelial and endothelial cells (Fig 11B and 11C), and the C/EBPβ motif located at -497 and -233 within the h*FCGRT* promoter in differentiated THP-1 cells (Fig 12B and 12C), could function as positive regulatory sequences in response to LPS or PMA stimulation. Each of the C/EBPβ sites in the h*FCGRT* promoter in THP-1 cells could function independently, because mutation of either of them did not interfere with a function of the other. In addition, C/EBPβ and C/EBPδ transcription factors were identified in the DNA-protein complexes formed between the C/EBPβ sites at -497 and -233 and nuclear

Sp1
-660 AAGCTTTGTAGCAGGAGTG**TGGGCGGGGGCTGGT**GCCTGGAGGGGCCTTCGAGTGAGGCAGCA
Sp1
-597 TTGTATGGGGA**CCATGT**GGTCACTGAAGTCCTAGCAGAAGGATGTGGTTTTTTTTTTTTTTG
CF1
-534 AGACGGAGTCTCGCTCTGTTGCCAGTCTGGAGTGCAG**TGGTGCAAT**CTCGGCTCACTGCGACC
C/EBPβ
-471 TCCACCTCCCGGGTTCAAGCAATTCTCCTGTCTCAGCCTCCCGAGTAGCTGGGACCACAGGCC

-408 CACCACCACGCTCAGCTAATTTTTGTATTATTAGTAGAGACGGGGGTGTCA**CCATAT**TGGCCA
CF1
-345 GGCTGGTCTCGAACTCCTGACCTCGTGATCCG**CCCGCC**TTGGCCTCCCAAAGTGCTGGGATTA
Sp1
-282 CAGGCG**TGAGTCA**CTGCGCCCGGCCCGCAGAAGGATATTAAAGAGCACCT**TGCAGGAAT**TTTT
Ap1 C/EBPβ
-219 AAGGGGATGCCGGGGGAAGGGCTTGAAGGAGGAACTAAGGAGGCTTCCAGAAGATTTTGATTA

-156 ACAGCGAGCGCTAGGGAAGGCCACGGGGATACAGAGGGGTAGGAGGAGGATCCAGAGACCTCC

-93 AAGTTGTGGACTCCGGAGGATCGAGTTCCTTTCCCAGTTTTGTCAAGAATTCCCTGAGCGGCA

-30 GACACCCTCAAAGTAAAAAACGGGAACCACAGAGAAGGAAAAAGAAGAACCACAAGCGTTTTG

AGAAACAAACAGGCTGGGC

Fig 13. Nucleotide sequence of the -660/+52 region of the h*FCGRT* promoter. Transcription factor binding sites involved in transcriptional regulation of the human *FCGRT* gene in Caco-2, Lu 106, HSKMEC, and PMA-differentiated THP-1 cell lines are underlined. The primary transcriptional start site (+1) is indicated by the curved arrow.

doi:10.1371/journal.pone.0135141.g013

extracts from differentiated THP-1 cells (Fig 6A and Fig 7). These nuclear proteins also interacted specifically with the C/EBPβ motif at -497 within the h*FCGRT* promoter in the stimulated epithelial cell lines (Fig 6B and 6C).

The Sp family transcription factors are important components of the eukaryotic cellular transcriptional machinery [43]. Ijang et al. [44] identified specific elements in the rat *Fcgrt* promoter that interacted with the members of the Sp1 family (Sp1, Sp2, Sp3) and drove promoter activity in intestinal cell lines. The Sp1 motif in the mouse *Fcgrt* promoter is critical in regulating the basal promoter function [45]. In this report, EMSA and supershift analyses showed that the binding motifs, functionally identified in the h*FCGRT* promoter, were able to specifically interact with their corresponding TFs (Sp1, Sp2, Sp3, c-Fos, c-Jun and YY1) suggesting a possible involvement of these transcription factors in the regulation of the basal expression of the human *FCGRT* gene (Figs 2–5 and S1–S4 Figs).

The results of the current study, concerning the contribution of response elements and transcription factors binding to these sequences in transcriptional regulation of the human *FCGRT* gene, were obtained from *in vitro* DNA binding experiments and promoter assays using mutant promoter luciferase constructs. Therefore, they may not accurately reflect the action in the endogenous promoter. Further studies on binding of transcription factors and their co-activators to the identified regulatory elements in the h*FCGRT* promoter in intact cells, as well as examining the interactions between these factors in the cellular environment and their involvement in the reorganization of chromatin structure should provide a foundation for understanding the mechanism of transcriptional regulation of the human *FCGRT* gene. The results presented in this work may serve as a basis for these studies.

Supporting Information

S1 Fig. Electrophoretic mobility shift assays with: probe Sp1-1+2 (C–E); probe Sp1-3 (C1–E1). The nuclear extracts from Caco-2 (C and C1), Lu 106 (D and D1), HUVEC (E and E1) cells were incubated with labeled probes in the absence of competitor (C–E1, lanes 2) or in the presence of a 100-fold molar excess of competitor: Sp1-1+2 (WT)–unlabeled wild-type probe Sp1-1+2 (C–E, lanes 3); Sp1-3(WT)–unlabeled wild-type probe Sp1-3 (C1–E1, lanes 3); mSp1-1+2 –unlabeled probe Sp1-1+2 containing mutation in the Sp1 sequence at positions -641 and -635 (C–E, lanes 4); mSp1-2 –unlabeled probe Sp1-1+2 containing mutation in the Sp1 sequence at position -635, (C–E, lanes 5); mSp1-3 –unlabeled probe Sp1-3 containing mutation in the Sp1 sequence at position -313 (C1–E1, lanes 4). Labeled probe Sp1-1+2(WT) alone (C–E, lanes 1); labeled probe Sp1-3(WT) alone (C1–E1, lanes 1). DNA-protein complexes were resolved on 5% non-denaturing polyacrylamide gels and analyzed in a phosphor imager (Typhoon 8600) using ImageQuant software (Molecular Dynamics). Positions of the specific DNA-protein complexes are indicated by arrows.
(TIF)

S2 Fig. Identification of transcription factors binding to the Sp1 sequences within the -660/-233 fragment of the h*FCGRT* promoter. Supershift experiments were performed by preincubating the nuclear extract from Caco-2 (C and C1), Lu 106 (D and D1) and HUVEC (E and E1) cells on ice for 1 h with 2 g of rabbit polyclonal antibodies specific for Sp family transcription factors or normal rabbit IgG prior to the addition of ^{32}P-labeled wild-type probe: Sp1-1+2(WT) (C–E), Sp1-3(WT) (C1–E1). Labeled probe alone (C–E1, lanes 1); labeled probe incubated with nuclear extract in the absence of antibodies (C–E1, lanes 2), in the presence of rabbit polyclonal antibodies: anti-Sp1 (C–E1, lanes 4), anti-Sp2 (C–E1, lanes 5), anti-Sp3 (C–E1, lanes 6), anti-AP-2 (C–E, lanes 7), normal rabbit IgG (C–E1, lanes 3). Shifted bands are

indicated with an asterisk. Results were analyzed by a phosphor imager.
(TIF)

S3 Fig. Characterization of the putative AP-1 binding site in the h*FCGRT* promoter by EMSA (C–E) and supershift analysis (C1–E1). Arrows indicate specific complex that formed during incubation of the wild-type AP1 ^{32}P-labeled probe–AP1(WT) with the nuclear extract from Caco-2 (C, lane 2), Lu 106 (D, lane 2) and HUVEC cells (E, lane 2). Competition experiments were performed in the presence of a 100-fold molar excess of the unlabeled probe AP1 (WT) (C–E, lanes 3) or in the presence of its mutated version in which the putative AP-1 binding site at position -276 was mutagenized–mAP1 (C–E, lanes 4). Supershift experiments were performed by preincubating the nuclear extracts with rabbit polyclonal anti-c-Fos antibody (C1–E1, lanes 3), anti-c-Jun antibody (C1–E1, lanes 4), normal rabbit IgG (C1–E1, lanes 5), prior to the addition of ^{32}P-labeled probe AP1(WT). Labeled probe AP1(WT) alone (C–E1, lanes 1); labeled probe AP1(WT) incubated with nuclear extract in the absence of antibodies (C1–E1, lanes 2). Position of the shifted complex is marked with an asterisk. Results were analyzed by a phosphor imager.
(TIF)

S4 Fig. Characterization of two potential CF1/YY1 regulatory motifs in the -660/-233 fragment of the h*FCGRT* promoter by EMSA (C–H) and supershift analysis (C1–H1). In EMSA, ^{32}P-labeled wild-type probes were incubated with nuclear extracts from Caco-2 (C and F, lanes 2), Lu 106 (D and G, lanes 2) and HUVEC cells (E and H, lanes 2) in the absence of competitor (C–H, lanes 2) or in the presence of a 100-fold molar excess of competitor: CF1-1 (WT)–unlabeled wild type probe CF1-1 (C–E, lanes 3); CF1-2 (WT)–unlabeled wild type probe CF1-2 (F–H, lanes 3); mCF1-1 –probe CF1-1 with mutation in the CF1 element at position -586 (C–E, lanes 4); mCF1-2 –probe CF1-2 containing mutation in the CF1 site at position -357 (F–H, lanes 4). Supershift experiments were performed by preincubating the nuclear extract with rabbit polyclonal anti-YY1 antibody (C1–H1, lanes 3), anti-Sp1 antibody (C1–E1, lanes 5), normal rabbit IgG (C1–H1, lanes 4), prior to the addition of ^{32}P-labeled wild-type probe: CF1-1(WT) (C1–E1); CF1-2(WT) (F1–H1). Labeled probes incubated with nuclear extract in the absence of antibodies (C1–H1, lanes 2). Labeled probe CF1-1(WT) alone (C–E1, lanes 1); labeled probe CF1-2(WT) alone (F–H1, lanes 1). Arrows indicate specific DNA-protein complexes and asterisk indicates the supershifted complex. Results were analyzed by a phosphor imager.
(TIF)

Acknowledgments

I am most grateful to Dr. D. Kłopotowska for technical assistance in the purification of promoter constructs, luciferase activity measurements, RT-PCR, and for assistance in preparing the figures. I thank M.Sc. J. Trynda for help in the statistical analysis of data from transient transfection assays. I also thank Wrocław Centre of Biotechnology, The Leading National Research Centre (KNOW) programme for years 2014–2018 for covering the publication fee.

Author Contributions

Conceived and designed the experiments: JEM. Performed the experiments: JEM. Analyzed the data: JEM. Contributed reagents/materials/analysis tools: JEM. Wrote the paper: JEM.

References

1. Simister NE, Rees AR. Isolation and characterization of an Fc receptor from neonatal rat small intestine. Eur J Immunol. 1985; 15:733–738. PMID: 2988974
2. Roberts DM, Guenthert M, Rodewald R. Isolation and characterization of the Fc receptor from the fetal yolk sac of the rat. J Cell Biol. 1990; 111:1867–1876. PMID: 2146275
3. Ahouse JJ, Hagerman CL, Mittal P, Gilbert DJ, Copeland NG, Jenkins NA, Simister NE. Mouse MHC class-I-like Fc receptor encoded outside the MHC. J Immunol. 1993; 151:6076–6088. PMID: 7504013
4. Story CM, Mikulska JE, Simister NE. A major histocompatibility complex class I-like Fc receptor cloned from human placenta: possible role in transfer of immunoglobulin G from mother to fetus. J Exp Med. 1994; 180:2377–2381. PMID: 7964511
5. Haymann JP, Levraud JP, Bouet S, Kappes V, Hagege J, Nguyen G, et al. Characterization and localization of the neonatal Fc receptor in adult human kidney. J Am Soc Nephrol. 2000; 11: 632–639. PMID: 10752522
6. Spiekermann GM, Finn PW, Ward ES, Dumont J, Dickinson BL, Blumerg RS, Lencer WI. Receptor-mediated immunoglobulin G transport across mucosal barriers in adult life: functional expression of FcRn in the mammalian lung. J Exp Med. 2002; 196:303–310. PMID: 12163559
7. Cianga P, Cianga C, Plamadeala P, Branisteanu D, Carasevici E. The neonatal Fc receptor (FcRn) expression in the human skin. Virchows Arch. 2007; 451:859–860. PMID: 17674040
8. Shah U, Dickinson BL, Blumberg RS, Simister NE, Lencer WI, Walker WA. Distribution of the IgG Fc receptor, FcRn, in the human fetal intestine. Pediatr Res. 2003; 53:295–301. PMID: 12538789
9. Powner MB, McKenzie JAG, Christianson GJ, Roopenian DC, Fruttiger M. Expression of neonatal Fc receptor in the eye. Invest Ophthalmol Vis Sci. 2014; 55:1607–1615. doi: 10.1167/iovs.13-12574 PMID: 24550358
10. Israel EJ, Taylor S, Wu Z, Mizoguchi E, Blumberg RS, Bhan A, Simister NE. Expression of the neonatal Fc receptor, FcRn, on human intestinal epithelial cells. Immunology 1997; 92:69–74. PMID: 9370926
11. Kobayashi N, Suzuki Y, Tsuge T, Okumura K, Ra C, Tomino Y. FcRn-mediated transcytosis of immunoglobulun G in human renal proximal tubular epithelial cells. Am J Physiol Renal Physol. 2002; 282: F358–F365.
12. Ghetie V, Ward ES. Multiple roles for the major histocompatibility complex class I-related receptor FcRn. Annu Rev Immunol. 2000; 18:739–766. PMID: 10837074
13. Antohe F, Radulescu L, Gafencu A, Ghetie V, Simionescu M. Expression of functionally active FcRn and differentiated bidirectional transport of IgG in human placental endothelial cells. Hum Immunol. 2001; 62:93–105. PMID: 11182218
14. Zhu X, Meng G, Dickinson BL, Li X, Mizoguchi E, Miao L, et al. MHC class I-related neonatal Fc receptor for IgG is functionally expressed in monocytes, intestinal macrophages, and dendritic cells. J Immunol. 2001; 166:3266–3276. PMID: 11207281
15. Vidarsson G, Stemerding AM, Stapleton NM, Spliethoff SE, Janssen H, Rebers FE, et al. FcRn: an IgG receptor on phagocytes with a novel role in phagocytosis. Blood 2006; 108: 3573–3579. PMID: 16849638
16. West APJ, Bjorkman PJ. Crystal structure and immunoglobulin G binding properties of the human major histocompatibility complex-related Fc receptor. Biochemistry 2000; 39: 9698–9708. PMID: 10933786
17. Szlauer R, Ellinger I, Haider S, Saleh L, Busch BL, Knöfler M, Fuchs R.. Functional Expression of the human neonatal Fc-receptor, hFcRn, in isolated cultured human syncytiotrophoblasts. Placenta 2009; 30:507–515. doi: 10.1016/j.placenta.2009.03.009 PMID: 19362735
18. Wani MA, Haynes LD, Kim J, Bronson CL, Chaudhury C, Mohanty S, et al. Familial hypercatabolic hypoproteinemia caused by deficiency of the neonatal Fc receptor, FcRn, due to a mutant $_{2}$-microglobulin gene. Proc Natl Acad Sci USA. 2006; 103:5084–5089. PMID: 16549777
19. Gan Z, Ram S, Vaccaro C, Ober RJ, Ward ES. Analyses of the recycling receptor, FcRn, in live cells reveal novel pathways for lysosomal delivery. Traffic 2009 May; 10(50:600. doi: 10.1111/j.1600-0854.2009.00887.x
20. Andersen JT, Daba MB, Sandle I. FcRn binding properties of an abnormal truncated analbuminemic albumin variant. Clin Biochem. 2010; 43:367–372. doi: 10.1016/j.clinbiochem.2009.12.001 PMID: 20006594
21. Akilesh S, Petkova S, Sproule TJ, Shaffer DJ, Christianson GJ, Roopenian D. The MHC class I-like Fc receptor promotes humorally mediated autoimmune disease. J Clin Invest. 2004; 113:1328–1333. PMID: 15124024

22. Yoshida M, Masuda A, Kuo TT, Kobayashi K, Claypool SM, Takagawa T, et al. IgG transport across mucosal barriers by neonatal Fc receptor for IgG and mucosal immunity. Springer Semin Immunopathol. 2006; 28:397–403. PMID: 17051393

23. Gosselin EJ, Bitsaktsis C, Li Y, Iglesias BV. Fc receptor-targeted mucosal vaccination as a novel strategy for the generation of enhanced immunity against mucosal and non-mucosal pathogens. Arch Immunol Ther Exp. 2009; 57:311–323.

24. Qiao SW, Kobayashi K, Johansen FE, Sollid LM, Andersen JT, Milford E, et al. Dependence of antibody- mediated presentation of antigen on FcRn. Proc Natl Acad Sci USA. 2008; 105:9337–9342. doi: 10.1073/pnas.0801717105 PMID: 18599440

25. Ye L, Liu X, Rout SN, Li Z, Yan Y, Lu L, et al. The MHC class II-associated invariant chain interacts with the neonatal Fc receptor and modulates its trafficking to endosomal/lysosomal compartments. J Immunol. 2008; 181:2572–2585. PMID: 18684948

26. Mikulska JE, Pablo L, Canel J, Simister NE. Cloning and analysis of the gene encoding the human neonatal Fc receptor. Eur J Immunogenet. 2000; 27:231–240. PMID: 10998088

27. Mikulska JE, Simister NE. Analysis of the promoter region of the human FcRn gene. Biochim Biophys Acta 2000; 1492:180–184. PMID: 11004487

28. Kieda C, Paprocka M, Krawczenko A, Zalecki P, Dupuis P, Monsigny M, et al. New human microvascular endothelial cell lines with specific adhesion molecules phenotypes. Endothelium 2002; 9:247–261. PMID: 12572856

29. Schreiber E, Matthias P, Müller MM, Schaffner W. Rapid detection of octamer binding proteins with „mini-extracts", prepared from a small number of cells. Nucleic Acids Res. 1989; 17:6419. PMID: 2771659

30. Bradford MM. A Rapid and sensitive method for the quantitation of microgram quantities of protein utilizing the principle of protein-dye binding. Anal Biochem. 1976; 72:248–254. PMID: 942051

31. Sato K, Nagai J, Mitsui N, Yumoto R, Takano M. Effects of endocytosis inhibitors on internalization of human IgG by Caco-2 human intestinal epithelial cells. Life Sci. 2009; 85: 800–807. doi: 10.1016/j.lfs.2009.10.012 PMID: 19879882

32. Kandil E, Noguchi M, Ishibashi T, Kasahara M. Structural and Phylogenetic analysis of the MHC class I-like Fc receptor gene. J Immunol. 1995; 154:5907–5918. PMID: 7538537

33. Gordon S, Akopyan G, Garban H, Bonavida B. Transcription factor YY1: structure, function, and therapeutic implications in cancer biology. Oncogene 2006; 25:1125–1142. PMID: 16314846

34. Lee JS, Galvin KM, Shi Y. Evidence for physical interaction between the zinc-finger factors YY1 and Sp1. Proc. Natl. Acad. Sci. USA. 1993; 90:6145–6149. PMID: 8327494

35. Seto E, Lewis B, Shenk T. Interaction between transcription factors Sp1 and YY1. Nature 1993; 365:462–464. PMID: 8003102

36. Berrier A, Siu G, Calame K. Transcription of a minimal promoter from the NF-IL6 gene is regulated by CREB/ATF and SP1 proteins in U937 promonocytic cells. J Immunol 1998; 161: 2267–2275. PMID: 9725220

37. Chen GK, Sale S, Tan T, Ermoian RP, Sikic BI. CCAAT/enhancer-binding protein β (Nuclear factor for interleukin 6) transactivates the human *MDR1* gene by interaction with a inverted CCAAT box in human cancer cells. Mol Pharmacol. 2004; 65:906–916. PMID: 15044620

38. Kwon HS, Kim MS, Edenberg HJ, Hur MW. Sp3 and Sp4 can repress transcription by competing with Sp1 for the core *cis*-elements on the human ADH5/FDH minimal promoter. J Biol Chem. 1999; 274:20–28. PMID: 9867805

39. Fry CJ, Farnham PJ. Context-dependent transcriptional regulation. J Biol Chem. 1999; 274:29583–29586. PMID: 10514422

40. Liu X, Ye L, Christianson GJ, Yang JQ, Roopenian DC, Zhu X. NF-B signaling regulates functional expression of the MHC class I-related neonatal Fc receptor for IgG via intronic binding sequences. J Immunol. 2007; 179:2999–3011. PMID: 17709515

41. Liu X, Ye L, Bai Y, Mojidi H, Simister NE, Zhu X. Activation of the JAK/STAT-1 signaling pathway by INF- can down–regulate functional expression of the MHC class I-related neonatal Fc receptor for IgG. J Immunol. 2008; 181:449–463. PMID: 18566411

42. Cervenak J, Doleschall M, Bender B, Mayer B, Schneider Z, Doleschall Z, et al. NFκB induces overexpression of bovine FcRn. A novel mechanism that further contributes to the enhanced immune response in genetically modified animals carrying extra copies of FcRn. Landeis Bioscience 2013; 5:860–871.

43. Kaczynski J, Cook T, Urrutia R. Sp1-and Krüppel-like transcription factors. Genome Biol. 2003; 4:206.1–206.8.

44. Jiang L, Wang J, Solorzano-Vargas RS, Tsai HV, Gutierrez EM, Ontiveros LO, et al. Characterization of the rat intestinal Fc receptor (*FcRn*) promoter: transcriptional regulation of *FcRn* gene by the Sp family of transcription factors. Am J Physiol Gastrointest Liver Physiol. 2004; 286:G922–G931. PMID: 15132949

45. Tiwari B, Junghans RP. Functional analysis of the mouse *Fcgrt* 5′ proximal promoter. Biochim Biophys Acta 2005; 1681:88–98. PMID: 15627500
